# Near‐Infrared Triggered Rechargeable Singlet Oxygen Battery for Sustained Phototherapy: Fast Charging and Long‐Lasting Discharging

**DOI:** 10.1002/advs.78284

**Published:** 2026-10-12

**Authors:** Yuxin Ren, Ling Li, Xinyi Zhang, Meiqi Li, Junqing Wang, Mengpan Zhang, Jian Wang, Yanli Tang

**Affiliations:** ^1^ Key Laboratory of Applied Surface and Colloid Chemistry Key Laboratory of Analytical Chemistry For Life Science of Shaanxi Province Ministry of Education School of Chemistry and Chemical Engineering Shaanxi Normal University Xi'an Shaanxi Province People's Republic of China

**Keywords:** anti‐tumor, aza‐BODIPY, NIR‐II, photodynamic therapy, singlet oxygen battery

## Abstract

Photodynamic therapy (PDT) for deep‐seated tumors is fundamentally constrained by limited tissue penetration, the ultrashort lifetime of singlet oxygen (^1^O_2_), and the lack of reusable oxygen delivery systems. Herein, we report a near‐infrared (NIR) triggered rechargeable “singlet oxygen battery” that enables sustained and cyclic ^1^O_2_ supply for enhanced phototherapy. A series of aza‐boron‐dipyrromethene (aza‐BODIPY) probes (NBDP, ABDP, and PBDP) are rationally engineered by integrating distinct ^1^O_2_ carriers, among which PBDP exhibits optimal performance. Upon 808 nm irradiation (5 min), PBDP nanoparticles (NPs), as the singlet oxygen battery, generate abundant ^1^O_2_ that is reversibly captured via [4+2] cycloaddition with 2‐pyridone to form endoperoxides (EPOs), while simultaneously enabling efficient photothermal conversion. After light cessation, stored ^1^O_2_ is thermally released from EPOs for up to 1 h, realizing sustained therapeutic action. Notably, the system supports multiple charging–discharging cycles with negligible performance decay. In vivo NIR‐II imaging reveals efficient tumor accumulation, leading to significant tumor suppression via synergistic sustained PDT/PTT (photothermal therapy) with excellent biocompatibility. This work provides a rechargeable ^1^O_2_ storage and release platform by integrating NIR activation, self‐heating‐assisted discharge, and cyclic phototherapeutic capability, offering a promising strategy to improve the efficiency and durability of phototherapy for tumor treatment.

## Graphical abstract

A near‐infrared‐triggered rechargeable singlet oxygen battery based on PBDP nanoparticles is developed. Upon 808 nm irradiation for 5 min, the system enables efficient singlet oxygen generation, reversible storage via cycloaddition, and sustained release for up to 1 h after light cessation. The rechargeable battery integrates photothermal conversion and supports synergistic sustained PDT/PTT for effective tumor therapy.

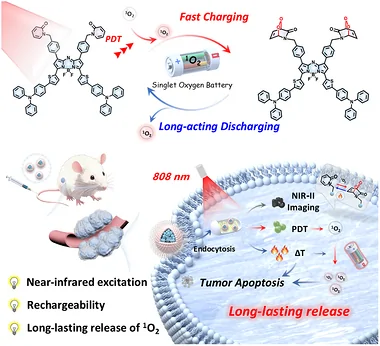

## 1 Introduction

The treatment of deep‐seated tumors remains a major challenge in cancer therapy [1, 2, 3]. As a noninvasive or minimally invasive strategy, phototherapy offers distinct advantages owing to its high spatial and temporal selectivity and low systemic toxicity [4, 5, 6, 7, 8]. Phototherapy mainly comprises photodynamic therapy (PDT) and photothermal therapy (PTT) [9, 10, 11]. In PDT, photosensitizers convert molecular oxygen (O_2_) into reactive oxygen species under light irradiation, particularly singlet oxygen (^1^O_2_), thereby inducing tumor cell death with high specificity [12, 13, 14, 15, 16]. However, PDT still faces several critical challenges in the treatment of deep‐seated tumors.

First, most conventional photosensitizers are activated by ultraviolet or visible light, which suffer from limited tissue penetration, thereby restricting their effectiveness in deep lesions [17, 18]. Therefore, it is imperative to develop photosensitive systems responsive to near‐infrared light to enhance tissue penetration. Second, ^1^O_2_ has an extremely short lifetime (10–320 ns) and is rapidly deactivated after light cessation, resulting in transient and unsustained therapeutic effects. To achieve sustained antitumor effects, prolonged irradiation is often required, which inevitably increases the risk of phototoxicity to surrounding normal tissues [19, 20, 21, 22]. Thus, developing systems capable of continuous ^1^O_2_ release after light cessation is essential for achieving sustained therapeutic outcomes. Third, most of the existing systems capable of ^1^O_2_ storage and release are single‐use and lose activity after single exposure, limiting their repeated use [23]. If controllable multiple cycles of ^1^O_2_ regeneration and release could be achieved after a single administration, it would enable repeated treatments from a single dose, thereby improving both therapeutic efficacy and clinical convenience. In summary, developing multifunctional systems integrating ^1^O_2_ generation, storage, sustained release, and recyclability is crucial for improving ^1^O_2_ utilization efficiency and enabling effective photodynamic therapy for deep‐seated tumors. However, achieving a rechargeable ^1^O_2_ platform with NIR activation, self‐heating‐assisted discharge, and tumor‐targeted phototheranostic capability remains challenging.

Based on above mentioned challenges, we developed a near‐infrared triggered rechargeable singlet oxygen battery for phototherapy. Specifically, we designed and synthesized a series of NIR‐II aza‐boron‐dipyrromethene (BODIPY) probes consisted of donors, aza‐BODIPY acceptors, π‐bridges, and different singlet oxygen carriers [24, 25, 26, 27] (Scheme 1a). BODIPY and its derivatives aza‐BODIPY compounds are widely employed in the biomedical field due to their excellent optical properties [28, 29, 30, 31]. The investigation about photophysical properties and structure‐property interactions of synthesized probes indicated that the probe PBDP modified with 2‐pyridone units was optimal in PDT. Then, the biocompatible nanoparticles PBDP NPs were obtained through self‐assembly with the amphiphilic polymer DSPE‐PEG_2000_ via nanoprecipitation [32, 33, 34, 35]. A near‐infrared light‐driven rechargeable singlet oxygen battery based on PBDP NPs thus was developed, demonstrating unique ^1^O_2_ storage and release abilities. Notably, under the irradiation of 808 nm laser only for 5 min, PBDP produce massive ^1^O_2_, and subsequently the 2‐pyridone group in PBDP form 2‐pyridone EPOs via [4+2] cycloaddition reaction, achieving efficient ^1^O_2_ storage due to super short distance [36, 37, 38, 39, 40, 41]. Additionally, the 2‐pyridone group in PBDP facilitates the creation of additional space for backbone distortion and rotor twisting. Consequently, PBDP NPs exhibit dissipation of excited‐state energy through nonradiative decay due to intramolecular free motion, producing photothermal effect (Scheme 1b). The residual thermal energy after removing the light source may promote the release of ^1^O_2_ from 2‐pyridone EPOs through thermal cyclization reduction, achieving a sustainable ^1^O_2_ release for 1 h. Additionally, this singlet oxygen battery can be rechargeable and keep the stable performance after multiple charging‐discharge. In vivo NIR‐II imaging demonstrated that the singlet oxygen battery could selectively accumulate at the tumor site through enhanced permeation and retention effects (EPR) and effectively kill cancer cells by durative PDT and PTT after irradiation (Scheme 1c) with high biosafety. This work paves the way to the development of a rechargeable and sustainable singlet oxygen battery platforms for efficient photodynamic antitumor therapy.

**SCHEME 1:**
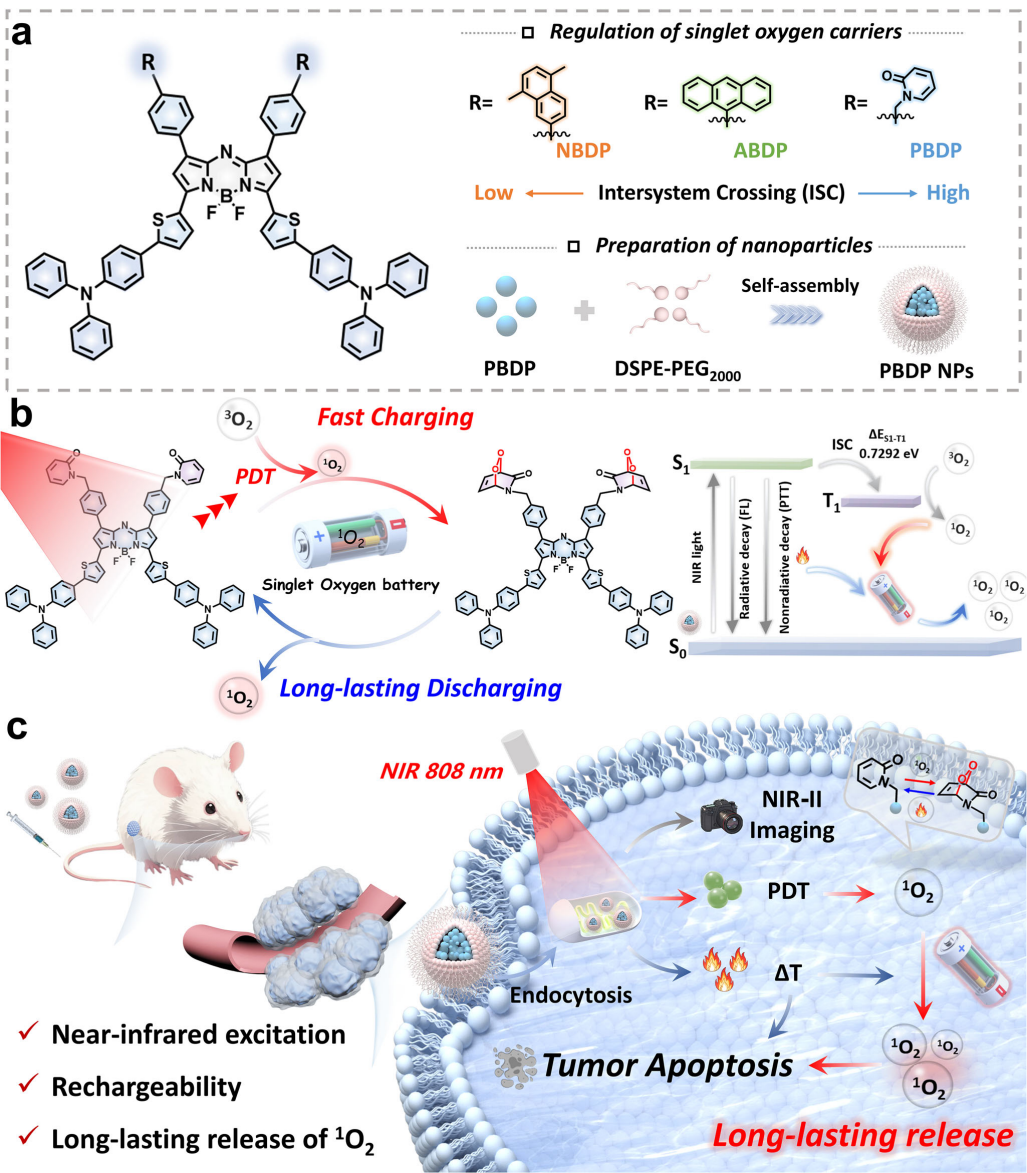
Schematic illustration of a rechargeable singlet oxygen battery with self‐heating triggered discharge for sustained PDT. (a) Molecular design and preparation of PBDP NPs. (b) Charging and discharging pathway of singlet oxygen battery and corresponding Jablonski Diagram. (c) Mechanism of PBDP NPs under NIR light irradiation for cancer treatment.

## 2 Results and Discussion

### 2.1 Design, Synthesis, and Characterization of NBDP, ABDP and PBDP Molecules

To construct NIR‐II rechargeable singlet oxygen battery, we designed and synthesized new probes of NBDP, ABDP, and PBDP through the singlet oxygen carrier modulation strategy. These probes consist of donors, aza‐BODIPY acceptors, π‐bridges and different singlet oxygen carriers. The triphenylamine donor was chosen as a strong electron donor [42], which has been widely used as an organic fluorophore to efficiently promote the emission redshift of probes. In addition, thiophene and benzene rings are used as π‐conjugated units and as electronic bridges for intramolecular charge transfer (ICT) conjugated to the acceptor [43]. The singlet oxygen carriers, 1.4‐Dimethylnaphthalene, anthracene, and 2‐pyridinone, are covalently linked with D‐π‐A type aza‐BODIPY, which can be reacted with ^1^O_2_ via [4+2] cycloaddition to give endoperoxides (EPOs) due to the presence of dienes in the six‐membered ring. EPOs is thermally unstable and prone to inverse Diels‐Alder reaction to regenerate ^1^O_2_ in high yields, enabling prolonged ^1^O_2_ release properties.

The synthetic routes of ABDP, NBDP, and PBDP are shown in Figure 1. The singlet oxygen carriers were obtained by Suzuki cross‐coupling and Nucleophilic substitution reaction. The NIR‐II aza‐BODIPY were synthesized through the classical synthetic route of O'Shea [44], which were fully characterized by ^1^H‐NMR, ^13^C‐NMR, and MALDI‐TOF‐MS (Figures S1‐S23). To investigate the structure‐property relationships of NBDP, ABDP, and PBDP (Figure 2a), theoretical calculations using density functional theory (DFT) were carried out. The geometries and frontier molecular orbitals of NBDP, ABDP, and PBDP are shown in Figure 2b. The three NIR‐II molecules have similar frontier molecular orbital density distributions. Their highest occupied molecular orbitals (HOMOs) are located mainly on the donor chain segments, with limited diffusion into the acceptor unit. In contrast, the lowest unoccupied molecular orbital (LUMOs) shows a small contribution from the central donor core, which is mainly located above the acceptor and π‐bridge units, further demonstrating the intrinsic intramolecular charge transfer (ICT) effect [45, 46]. The energy gaps of the three molecules decrease from 1.62 and 1.61 to 1.60 eV, indicating a gradual increase in their D‐A strength. In particular, PBDP shows a more distorted conformation and enhanced ICT effect, which not only hinders the intermolecular π‐π interactions for robust emission, but also induces red‐shifted emission.

**FIGURE 1:**
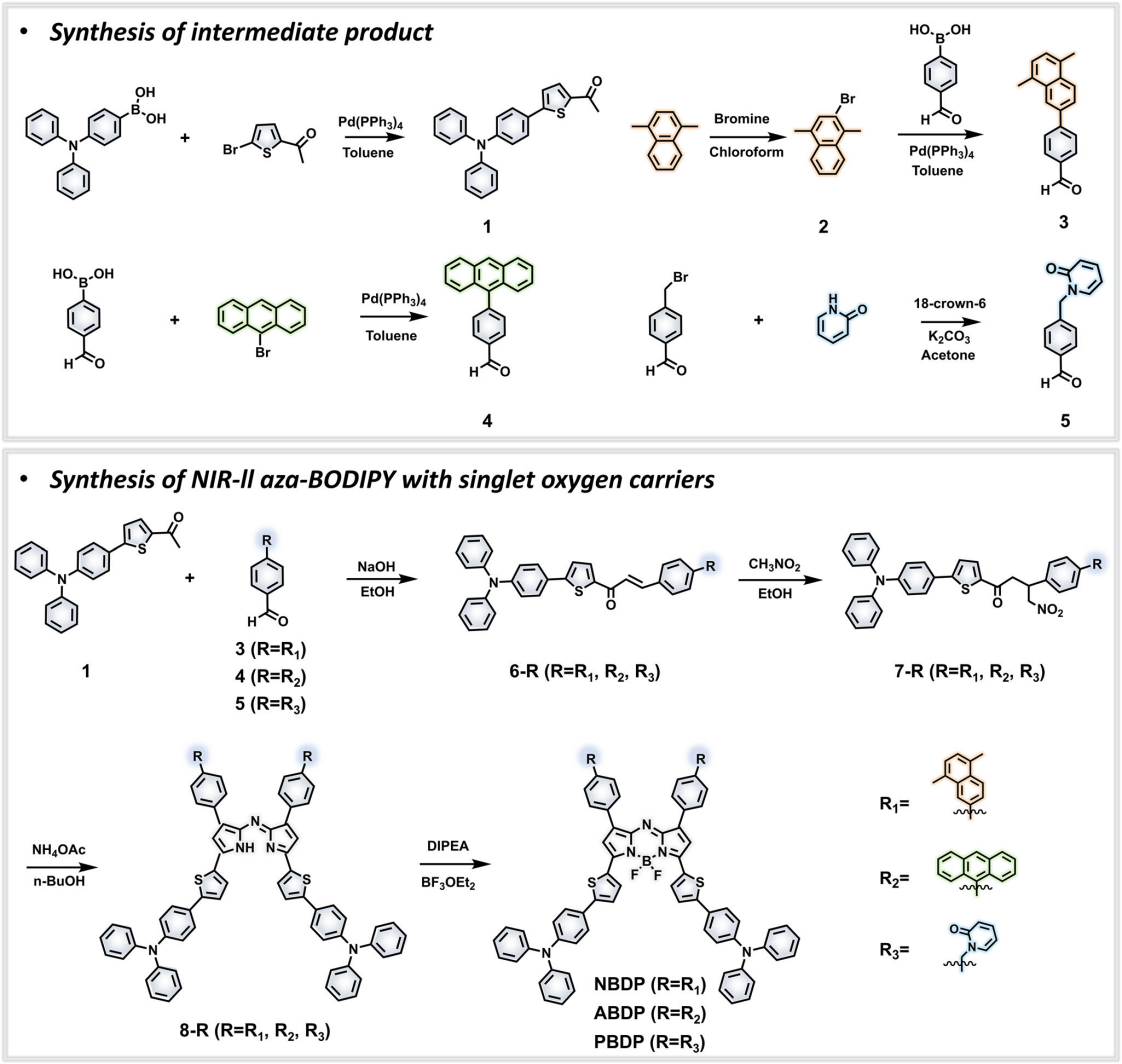
Synthesis route of NBDP, ABDP, and PBDP.

**FIGURE 2:**
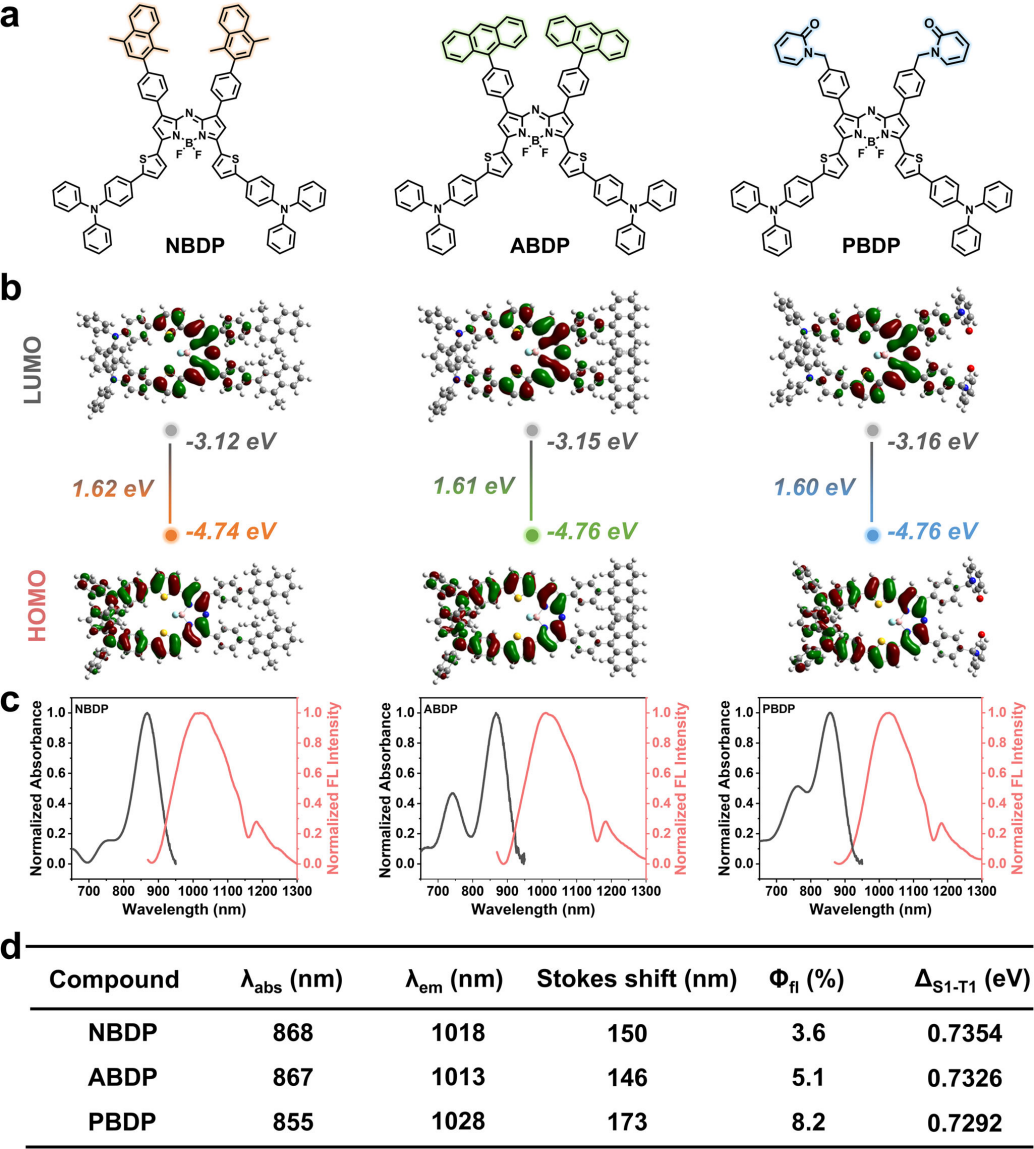
Molecular structures, theoretical calculations, and photophysical characterizations of aza‐BODIPY fluorophores. (a) Chemical structures of NBDP, ABDP, and PBDP. (b) Energy levels of LUMOs and HOMOs for the S_0_ states of molecules. (c) Normalized absorption spectra and emission spectra of the molecules in DCM. (d) photosensitizing properties of NBDP, ABDP, and PBDP.

Next, the photophysical properties of probes were studied in dichloromethane (DCM). As shown in Figure 2c,d, NBDP, ABDP, and PBDP show typical NIR‐absorbing capabilities with maximal absorbances at 868, 867, and 855 nm, respectively. Under 808 nm light excitation, the emission peaks of NBDP, ABDP, and PBDP are characterized to be at 1018, 1013, and 1028 nm, with stokes shifts are 150, 146 and 173 nm, respectively. The fluorophore with large stokes shift is not easy to be reabsorbed by adjacent molecules, so PBDP shows the advantages of a stronger signal and higher stability in imaging. The NIR‐II fluorescence quantum yields (QYs) of NBDP, ABDP, and PBDP were calculated as 3.6%, 5.1%, and 8.2% (Figure S24), respectively. The energy difference ΔE_S1‐T1_ between the lowest singlet excited state (S_1_) and the lowest triplet excited state (T_1_) of molecules were further calculated, which showed that NBDP, ABDP, and PBDP displayed ΔE_S1‐T1_ values of 0.7354, 0.7362, and 0.7292 eV, respectively, suggesting a progressive increase in the intersystem crossing (ISC) process photodynamic performance. The above data indicate that the PBDP molecules modified by 2‐pyridone group exhibit longer emission wavelength, larger Stokes shift, higher quantum yield, and stronger intersystem crossing performance, enabling robust potential for deep tissue PDT. Thus, PBDP was selected as a ^1^O_2_ carrier agent for the following investigation. Considering that the ^1^O_2_ storage and release behavior is critical for constructing a rechargeable singlet oxygen battery, the storage‐related performance of NBDP, ABDP, and PBDP was further investigated using SOSG assays. As shown in Figures S25 and S26, PBDP exhibited the strongest SOSG fluorescence enhancement under 808 nm irradiation and the most pronounced dark‐discharge ^1^O_2_ release after light cessation compared with ABDP and NBDP. These results demonstrate that the introduction of 2‐pyridone units endows PBDP with superior ^1^O_2_ storage and sustained‐release capability, supporting its selection for the construction of the rechargeable ^1^O_2_ battery platform.

### 2.2 Preparation and Characterization of PBDP NPs

Furthermore, we prepared PBDP NPs that served as singlet oxygen battery by self‐assembly of PBDP with DSPE‐PEG_2000_ (Figure 3a) [47]. As shown in Figure 3b, the average hydrated diameter of the prepared PBDP NPs was about 112 nm. Scanning electron microscope (SEM) images of the nanoparticles showed spherical morphology, and the liposome wrapped in the outer layer was clearly visible (Figure 3c). Also, the zeta‐potential value was measured as −33.1 ± 1.0 mV for PBDP NPs. The negative charge prolongs liposome circulation and reduces clearance [48]. To further evaluate the colloidal stability of PBDP NPs under physiologically relevant conditions, the changes in hydrodynamic size, PDI, and zeta potential were monitored in PBS and serum‐containing culture medium for 72 h. As shown in Figures S27 and S28, PBDP NPs exhibited negligible changes in particle size, narrow PDI distribution, and stable surface charge during the monitoring period, indicating excellent dispersion stability and resistance to aggregation in complex biological environments. Next, we investigated the optical properties of the nanoparticles. As shown in Figure S29, the maximal absorption wavelength of PBDP NPs was at 875 nm, and the maximal fluorescence emission wavelength of NPs was at 1110 nm, which further demonstrated the successful preparation of nanoparticles. The concentration of PBDP in PBDP NPs was quantified based on a pre‐established calibration curve at 855 nm by UV‐Vis spectroscopy (Figure S30). The QY of PBDP NPs was calculated as 0.3% (Figure S31). Subsequently, we investigated the photodynamic properties of PBDP NPs and the pristine PBDP molecule using 2',7'‐dichlorodihydrofluorescein (DCFH) as a total ROS probe. Surprisingly, the fluorescence emission intensity of 2',7'‐dichlorofluorescein (DCF) at 525 nm in the PBDP NPs group was significantly enhanced upon irradiation, 43‐fold higher than that of the blank control group, indicating its excellent ROS generation ability (Figure 3d,e). In contrast, the photodynamic performance of the pristine PBDP molecule was 27‐fold that of the blank group (Figure S32), highlighting the remarkable improvement in ROS production efficiency after nano‐formulation.

**FIGURE 3:**
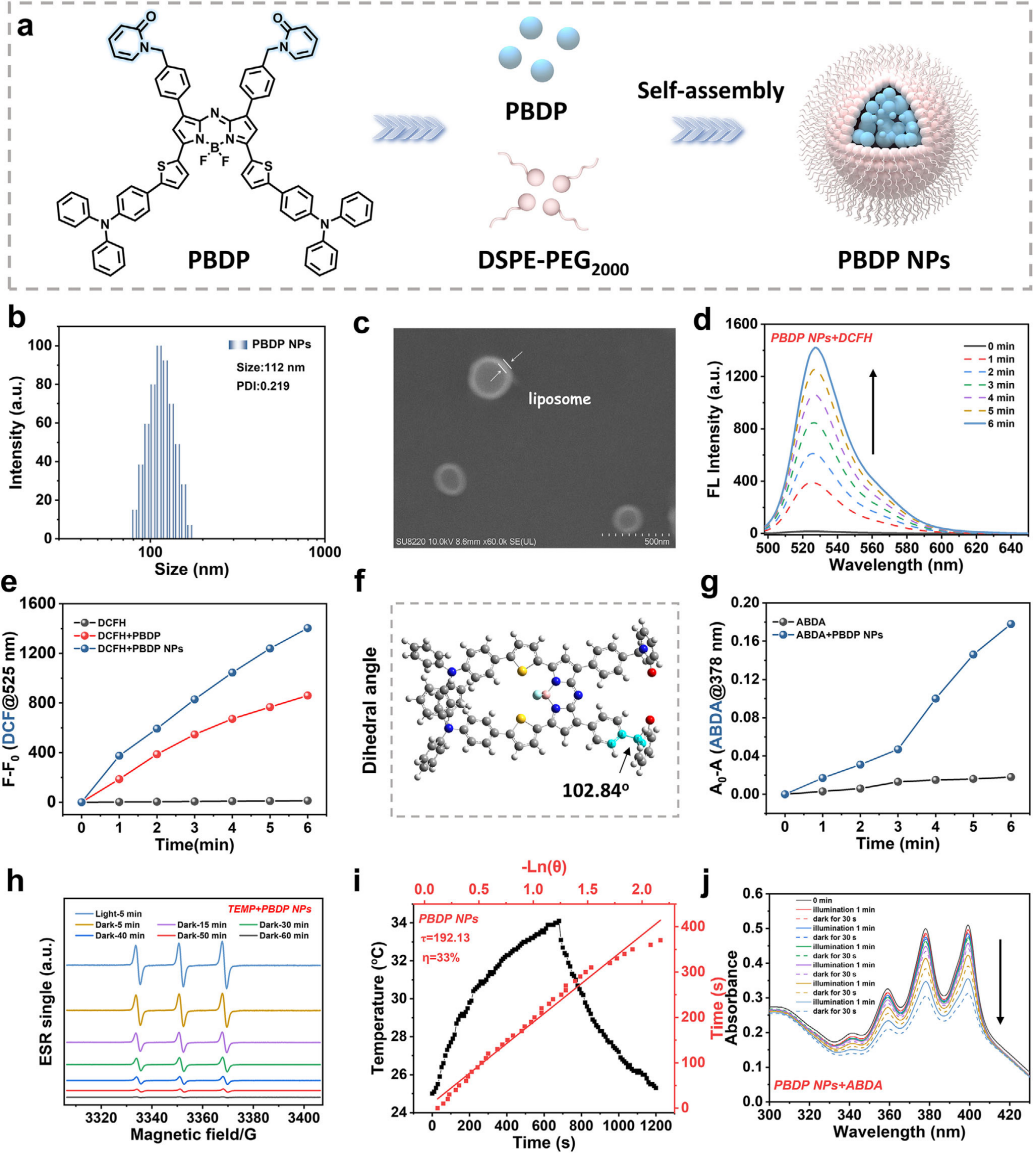
Preparation of nanoparticles and photodynamic properties of PBDP NPs. (a) Schematic illustration of the PBDP NPs preparation. (b) DLS data of PBDP NPs. (c) SEM image of nanoparticles. (d) The fluorescence spectra of DCF in the presence of PBDP NPs after irradiation at different times. (e) Changes in the fluorescence intensity of the DCF in the presence of PBDP or PBDP NPs upon 808 nm irradiation. (f) Optimized geometries and dihedral angles of PBDP. (g) Changes in the absorbance of ABDA probe in the presence of PBDP NPs upon 808 nm irradiation. (h) Time‐dependent ESR spectra of TEMP in the presence of PBDP NPs after 5 min of light irradiation and during the subsequent dark discharge period (5, 15, 30, 40, 50, and 60 min). (i) Self‐heating properties of PBDP NPs. (j) Degradation of ABDA in the presence of PBDP NPs with or without irradiation.

To elucidate the mechanism of explosive ROS generation by PBDP NPs, we performed theoretical calculations to analyze their geometric configurations and electron distributions. The calculation results demonstrated that the dihedral angle of the 2‐pyridone moiety in PBDP reached 102.84° (Figure 3f), indicating a significant distortion of this structural segment. This twisted configuration can effectively inhibit the π‐π stacking interaction between molecules, thereby promoting the transition process, thereby significantly enhancing the reactive oxygen species generation ability of nanoparticles [49, 50, 51, 52, 53]. Then, the ^1^O_2_ generated by PBDP NPs was explored using 9,10‐anthracenediyl‐bis(methylene)dimalonic acid (ABDA) as ^1^O_2_ probe. As shown in Figure 3g, under irradiation for 6 min, the absorbance of ABDA solution containing PBDP NPs showed an obvious decreasing trend, indicating that PBDP NPs have excellent ^1^O_2_ production ability. As shown in Figure 3h, the characteristic 1:1:1 triplet ESR signal of TEMP‐^1^O_2_ was clearly observed after irradiation of PBDP NPs, confirming efficient ^1^O_2_ generation. After switching off the laser, the ESR signal persisted throughout the dark period, directly demonstrating the sustained release of stored ^1^O_2_ and the rechargeable singlet oxygen battery behavior of PBDP NPs.

As shown in Figure 3i, the photothermal conversion efficiencies (PCE) of PBDP NPs was measured under 808 nm laser irradiation as 33.3%. The above results indicated that PBDP NPs exhibit favorable self‐heating properties, enabling their ^1^O_2_ release in singlet oxygen battery. Furthermore, by simultaneously monitoring the ^1^O_2_ generation and temperature variations during alternating cycles of illumination and darkness, the dynamic interplay and transition mechanism between the photodynamic and photothermal effects of PBDP NPs were further elucidated. As shown in Figure 3j and Figure S33, when PBDP NPs were added, the absorbance of ABDA decreased after 1 min irradiation, and further decreased under the subsequent 30 s dark conditions, which suggested that even in the absence of light, PBDP NPs can still release the stored ^1^O_2_. Commercially available photosensitizers (MB and ICG) served as controls. There was no change detected in their ABDA solutions in the dark (Figure S34), demonstrating the inability of unmodified photosensitizers to generate ^1^O_2_ in the absence of light. These results further verify that the 2‑pyridone groups enable sustained ^1^O_2_ release under dark conditions. The continuous ^1^O_2_ generation ability of PBDP NPs was also evaluated by monitoring the decrease in absorbance of 1,3‐diphenylisobenzofuran (DPBF) at 414 nm due to oxidation reaction. As shown in Figure S35, the absorbance of DPBF at 414 nm decreased under both light and dark conditions, indicating that PBDP NPs can continuously release singlet oxygen in the dark environment, and its performance is better than similar photosensitizers. To clarify the contribution of the singlet oxygen battery effect, a structurally matched control molecule (BBDP) was designed by replacing the 2‐pyridone unit in PBDP with a phenyl ring while retaining the aza‐BODIPY core and photothermal framework (Figures S36‐S40). BBDP was further formulated into nanoparticles (BBDP NPs) using DSPE‐PEG_2000_. Unlike PBDP NPs, BBDP NPs retained photodynamic and photothermal activities while lacking reversible ^1^O_2_ capture and release capability (Figure S41), thus serving as a reliable control for distinguishing the therapeutic enhancement derived from the rechargeable singlet oxygen battery function from conventional PDT/PTT effects.

### 2.3 “Charging” and “Discharging” Mechanism of Singlet Oxygen Battery

Due to excellent PDT efficacy of PBDP NPs, the “charging” and “discharging” mechanism of PBDP NPs as the rechargeable singlet oxygen battery was investigated. The mechanism diagram of the singlet oxygen battery is shown in Figure 4a. Under 808 nm laser irradiation, the 2‐pyridone group in PBDP can undergo a [4+2] cycloaddition reaction with ^1^O_2_ to form an unstable internal peroxide, thereby realizing the storage of ^1^O_2_. The internal peroxide structure can further undergo a reverse reaction of reconverting into 2‐pyridone groups, and thus releasing the stored ^1^O_2_. We used mass spectrometry to further characterize the “charging” and “discharging” processes of singlet oxygen batteries. As shown in Figure 4b–g, before laser irradiation, mass spectra showed that the molecular weight of PBDP itself was 1210 (m/z). After irradiation with light at 808 nm for 5 min, a new and strong molecular weight peak at 1274 (m/z) appeared on the mass spectrum, which belongs to the molecule PBDP‐EPO generated after capturing ^1^O_2_ by PBDP. These results indicate the “charging” of singlet oxygen battery is successful. After standing in the dark for 5 min, an additional peak at 1242 (m/z) was detected, which corresponded to the single pyridone (PBDP‐EPO‐INT+H), confirming that a stepwise reverse reaction process occurred in the singlet oxygen battery. After 15 min, the peak at 1274 (m/z) disappeared, and the peaks at 1242 (m/z) and 1210 (m/z) were generated, which were attributed to the molecular peak of PBDP‐EPO‐INT+H and PBDP+H, respectively, indicating that singlet oxygen battery is in the process of “discharge”. After 30 min, the relative proportion of the molecular peak at 1242 (m/z) decreased, and the molecular peak at 1210 (m/z) increased, indicating that the “discharging” process was further carried out. Finally, after 60 min, the peak at 1242 (m/z) disappeared completely, and only the molecular peak at 1210 (m/z) was left, indicating that the internal peroxide was converted into 2‐pyridone group completely, and the “discharging” of singlet oxygen battery was accomplished. The above results show that when the singlet oxygen battery is charged for 5 min, and it can continuously release ^1^O_2_ for up to 60 min, demonstrating the super ability of rapid storage and long‐lasting release of ^1^O_2_.

**FIGURE 4:**
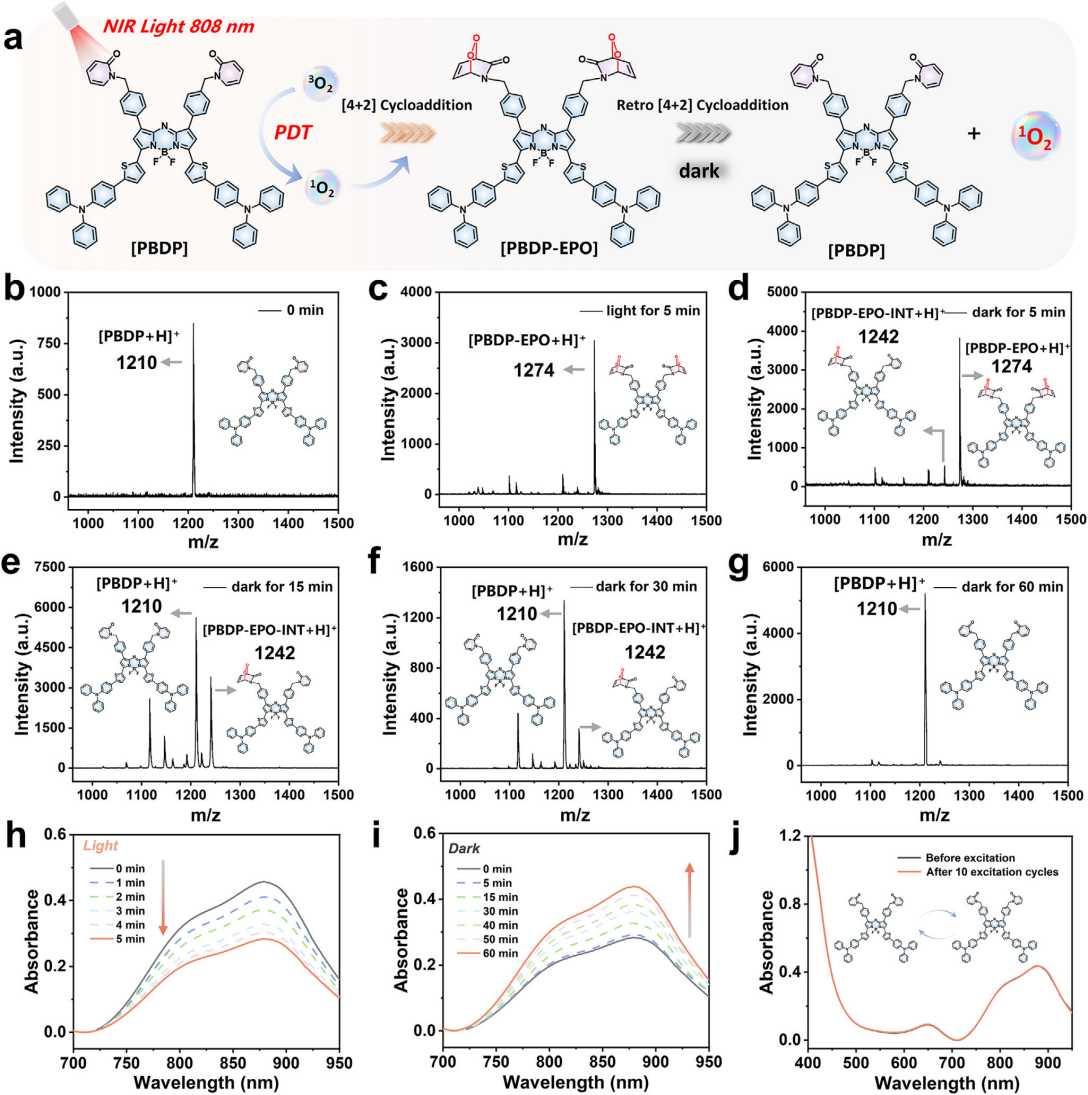
Charging and discharging performance of singlet oxygen battery. (a) The mechanism of PBDP capture and release ^1^O_2_. (b‐g), MALDI‐TOF spectrum of PBDP before, after 808 nm laser irradiation for 5 min, and then stored in the dark for 60 min at room temperature (25°C). (h)The absorption spectra changes of PBDP NPs (40 µg/mL) in 5 min after 808 nm laser irradiation. (i) The absorption spectra changes of PBDP NPs in the dark for 60 min. (j) The absorption spectra changes of PBDP NPs before and after 10 excitation cycles.

Then, to further verify the “charging” and “discharging” performance of the battery, the absorption spectra of PBDP NPs before and after illumination were measured. PBDP NPs showed the maximal absorption peak at 875 nm, and the absorbance decreased significantly after 5 min of 808 nm laser irradiation (Figure 4h), which may result from the fast transition from PBDP to PBDP‐EPO. Over the next 60 min in the dark, the absorbance gradually and slowly recovered to its initial intensity (Figure 4i), indicating a recovery from PBDP‐EPO to PBDP. This result also proved the reversible conversion between “charging” and “discharging”. Quantitative analysis revealed that the normalized absorbance at 878 nm decreased continuously during the 5 min irradiation period, corresponding to an apparent conversion efficiency of 37% (Figure S42a,b). After light removal, the absorbance gradually recovered in the dark, with the recovery percentage reaching 90% within 60 min (Figure S42c), indicating efficient discharge of stored singlet oxygen. To further verify the generation and sustained dark release of ^1^O_2_, SOSG fluorescence measurements were performed. As shown in Figure S43, the fluorescence intensity of SOSG at 535 nm increased markedly during 5 min of irradiation in the presence of PBDP NPs. After cessation of irradiation, the SOSG fluorescence of the PBDP NPs system continued to increase progressively over the subsequent 60 min dark‐discharge period. These results provide singlet oxygen‐selective and time‐dependent evidence supporting the photoinduced generation and subsequent sustained dark release of ^1^O_2_ by PBDP NPs. Surprisingly, there was no significant change in the absorption of PBDP NPs after 10 excitation cycles (irradiated for 5 min under illumination and placed in dark for 60 min as a cycle) (Figure 4j). Moreover, PBDP NPs maintained over 90% recovery after ten consecutive charging–discharging cycles (Figure S44), indicating that the singlet oxygen battery has significant reversibility and stability.

### 2.4 Effect of Self‐Heating on the Discharge Process

Due to the nanoparticles' photothermal effect, we speculate the light‐induced heat drives endoperoxide reverse reactions to release ^1^O_2_. To verify the speculation, ABDA was used as a ^1^O_2_ probe for the detection of ^1^O_2_ under different temperature and stirring conditions. First, PBDP NPs and ABDA mixed solution was irradiated under 808 nm laser for 5 min, followed by incubation for 1 h at 4°C. Figure 5a shows ABDA's characteristic peaks decreased significantly under light illumination, confirming efficient ^1^O_2_ generation from PBDP NPs. However, no absorption decrease occurred when the solution was kept in the dark. As a control, the absorption of ABDA alone remained unchanged during the whole experiments (Figure 5b,f,j,n). These results indicated low temperature suppressed ^1^O_2_ release due to the limited self‐relaxation heat preventing endoperoxide bond cleavage (Figure 5c,d). To confirm the effect of heat on ^1^O_2_ release, we conducted the experiment by stirring the solution at 25°C to accelerate the heat dissipation, avoiding heat energy being captured by the nanoparticles. It is observed that ABDA degradation was obvious after light illumination for 5 min (Figure 5e), however, only weak ABDA degradation was measured when incubated in dark under stirring (Figure 5g), which was attributed to heat diffusion via stirring, limiting endoperoxide conversion and ^1^O_2_ release (Figure 5h). Notably, when the PBDP NPs and ABDA mixed solution was irradiated for 5 min and then kept in dark for 1 h at 25°C without stirring (Figure 5i,k), ABDA peaks significantly decreased not only after illumination but also during incubation in dark (Figure 5l). Considering that the physiological temperature of living organisms is 37°C, we irradiated the mixture of PBDP NPs and ABDA at 37°C without stirring for 5 min, followed by incubation in the dark for 1 h (Figure 5m,o). The results showed that the ABDA peak not only decreased significantly after irradiation, but also exhibited a further marked reduction during dark incubation compared to the condition at 25°C. This is attributed to the accelerated cleavage of the oxygen bridge bond at 37°C, which promoted the release of ^1^O_2_ (Figure 5p). These results indicate that the heat generated by PBDP NPs themselves can effectively trigger the conversion of endoperoxide to pyridone, enabling sustained ^1^O_2_ production without the need for external light irradiation. It is worth mentioning that the photothermal effect occurs when PBDP NPs is irradiated, which induces the ^1^O_2_ release. However, due to character of the fast production and slow release of ^1^O_2_, the PBDP NPs primarily exhibit charged properties during light exposure. To further evaluate the influence of photothermal heating on the sustained release of ^1^O_2_, the ABDA assay was performed at 43°C, corresponding to the estimated local temperature after a photothermal increase of approximately 6°C. As shown in Figure S45, The ABDA absorption at 378 nm decreased during 5 min of laser irradiation and continued to decrease during the subsequent 60 min dark period. In contrast, ABDA alone remained nearly unchanged under the same conditions. These results demonstrate that PBDP NPs retain sustained ^1^O_2_ release capability at an elevated temperature relevant to their photothermal effect. This validates PBDP NPs as a promising “singlet oxygen battery” for applications in living organisms.

**FIGURE 5:**
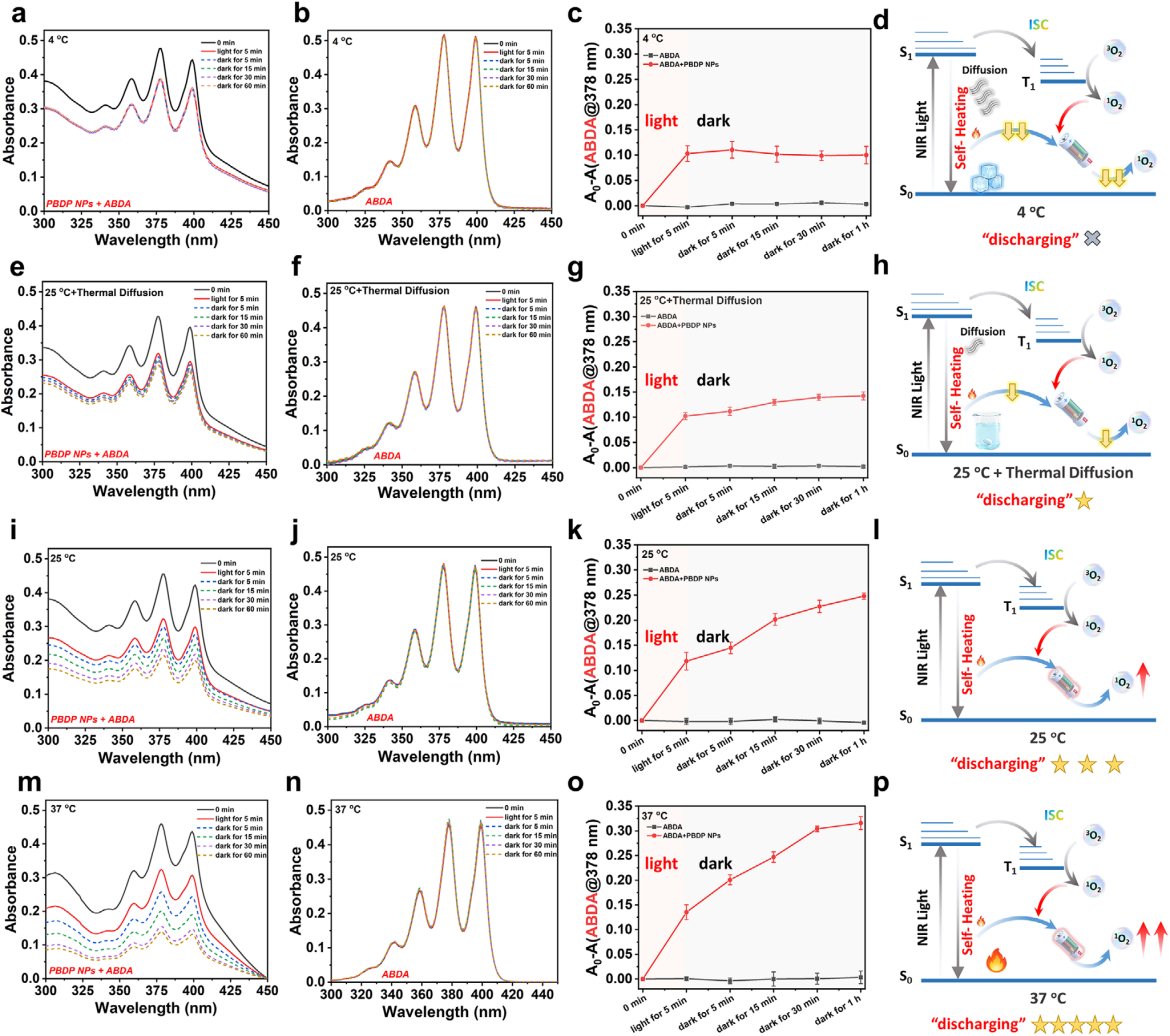
The effect of self‐heating on the discharge process. (a‐d) Degradation of ABDA by PBDP NPs (40 µg/mL) with or without light irradiation at 4°C and corresponding Jablonski diagram. (e‐h) Degradation of ABDA by PBDP NPs with or without light irradiation at 25°C with thermal diffusion and corresponding Jablonski diagram. (i‐l) Degradation of ABDA by PBDP NPs with or without light irradiation at 25°C and corresponding Jablonski diagram. (m‐p) Degradation of ABDA by PBDP NPs with or without light irradiation at 37°C and corresponding Jablonski diagram. Data are presented as the mean ± SD (n = 3).

### 2.5 Intracellular Behaviors of PBDP NPs

The uptake of PBDP NPs in MCF‐7 cells was preliminarily confirmed using NIR‐II imaging system (Figure S46). At 3 h, a distinct fluorescence signal of PBDP NPs was observed in the cells via imaging, and the fluorescence intensity reached its maximum at 12 h, indicating efficient uptake of PBDP NPs by MCF‐7 cells. Subsequently, we prepared Nile Red and PBDP together with DSPE‐PEG to form PBDP‐N NPs. Nile Red was used to track the distribution pathway of the nanoparticles within cells. Colocalization fluorescence imaging revealed a high degree of overlap between the green fluorescence of Mito‐tracker and the red fluorescence of PBDP‐N NPs, with a Pearson's correlation coefficient of 0.73 (Figure 6a,b). The results demonstrated that PBDP NPs could be internalized via the endocytic pathway and distributed in mitochondria.

**FIGURE 6:**
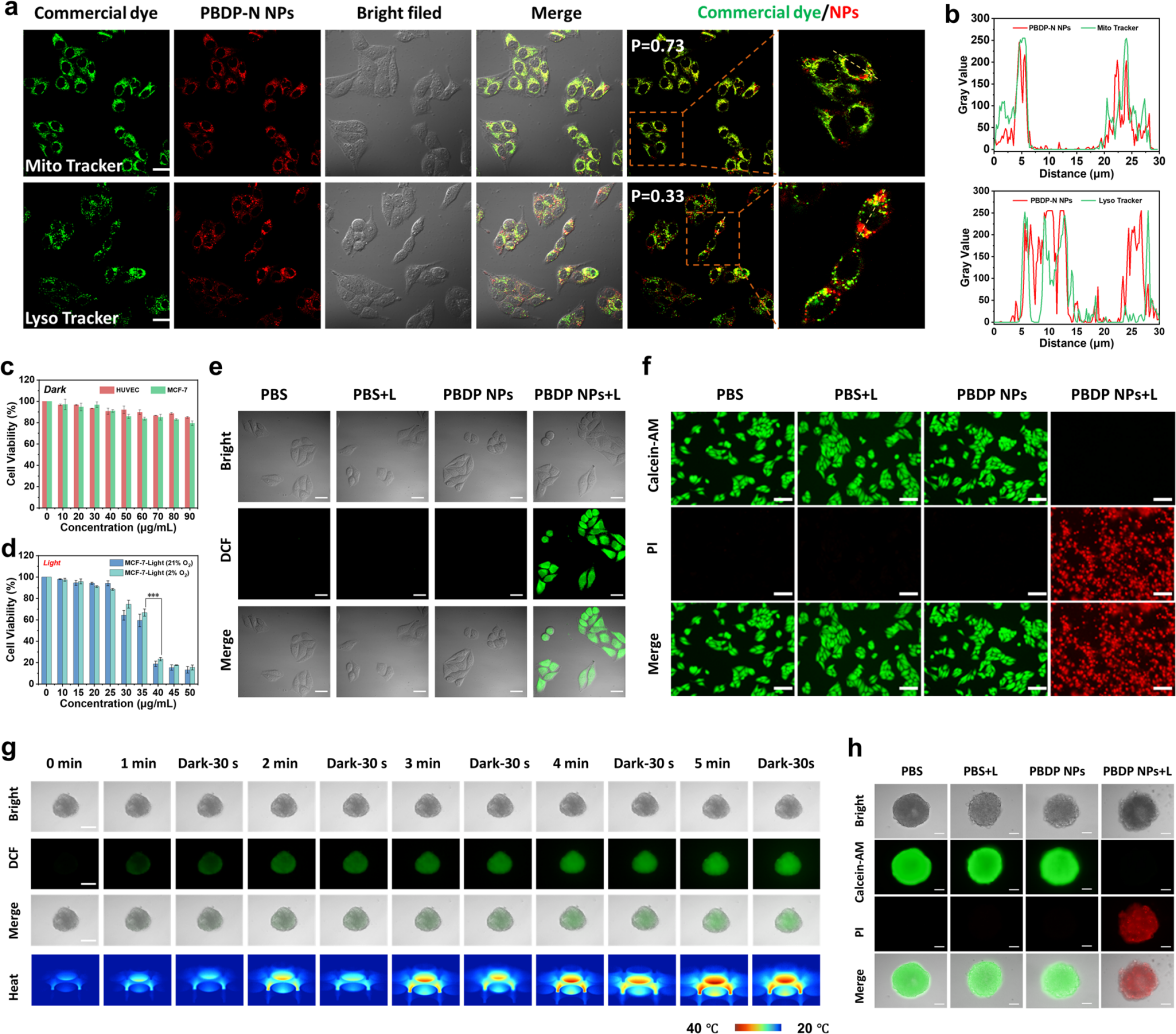
The intracellular behaviors of singlet oxygen battery. (a) Confocal laser scanning microscopy images of MCF‐7 cells after treatment with PBDP NPs for 12 h and stained by Mito tracker or Lyso tracker. (b) Linear analysis of selected regions in CLSM images of (a). (c) Cell viability of HUVEC cells or MCF‐7 cells after incubation with PBDP NPs of different concentrations without 808 nm laser irradiation. (d) Cell viability of MCF‐7 cells after incubation with different concentrations of PBDP NPs under normoxic and hypoxic conditions, followed by 808 nm laser irradiation (0.5 W/cm^2^, 5 min). (e) Confocal laser scanning microscopy images of MCF‐7 cells stained with DCFH‐DA to detect ROS generation following different treatments. Scale bar = 30 µm. (f) Calcein‐AM (green, live cells)/propidium iodide (PI; red, dead cells) staining of MCF‐7 cells treated with PBDP NPs without or with laser irradiation (scale bar: 100 µm). (g) The ROS generation inside 4T1 MCTSs without or with irradiation after 12 h of incubation with PBDP NPs and corresponding photothermal temperature (scale bar: 200 µm). (h) Calcein‐AM (green, live cells)/propidium iodide (PI; red, dead cells) staining of 4T1 MCTSs treated with PBDP NPs without or with laser irradiation (scale bar: 100 µm). Data are presented as the mean ± SD (n = 3). (***) p < 0.001.

### 2.6 In Vitro Cell Biocompatibility and Phototoxicity

As phototherapeutic agents, low cytotoxicity and acceptable biocompatibility are key for biomedical applications. Therefore, the biocompatibility of the PBDP NPs on HUVEC cells and MCF‐7 cells was examined using the methyl thiazolyl tetrazolium (MTT) method. As shown in Figure 6c, it was observed that when PBDP NPs were used even at a high concentration of 90 µg/mL, the survival rate of both HUVEC and MCF‐7 cells was still higher than 80%. These results indicated that the PBDP NPs had great biocompatibility.

After irradiation with 808 nm laser (0.5 W/cm^2^) for 5 min, the survival rates of MCF‐7 cells decreased in a concentration‐dependent manner (Figure 6d). Notably, a sharp decline in cell viability was observed when the concentration increased from 35 to 40 µg/mL. This nonlinear dose response may be attributed to a threshold effect, where at 40 µg/mL, the production of ROS likely exceeds the antioxidant capacity of the cells. At this concentration, the survival rate of MCF‐7 cells treated with PBDP NPs was approximately 19%. The excellent antitumor ability of PBDP NPs is attributed to the long‐term sustained release of ^1^O_2_ triggered by the self‐heating effect after irradiation, as well as their own photothermal properties. The contribution of ROS‐mediated photodynamic effects to the therapeutic activity of PBDP NPs was further evaluated using vitamin C (Vc) as a ROS scavenger. Under NIR irradiation, PBDP NPs induced concentration‐dependent photocytotoxicity, whereas the addition of Vc significantly restored cell viability (Figure S47), confirming that ROS‐mediated photodynamic damage plays a key role in the antitumor effect of PBDP NPs. Subsequently, we used DCFH‐DA as a probe to detect intracellular ROS levels. Strong green DCF fluorescence was observed in the experimental group of PBDP NPs with irradiation, indicating that PBDP NPs could produce a large amount of ROS in living cells under 808 nm laser irradiation, which effectively induced tumor cell death (Figure 6e and Figure S48). To simulate the hypoxic tumor microenvironment, cells were cultured under low‐oxygen conditions (2% O_2_), and similar results were obtained. This indicates that PBDP nanoparticles can maintain their photodynamic effect and effectively kill tumor cells even in a hypoxic environment (Figure S49). Furthermore, flow cytometry analysis revealed that PBDP NPs + laser treatment induced markedly enhanced DCF fluorescence under both normoxic and hypoxic conditions (Figure S50). These results demonstrate that PBDP NPs maintain efficient ROS generation even under oxygen‐deficient conditions. In addition, calcein‐AM/propidium iodide (PI) staining further confirmed the effective tumor cell‐killing ability of PBDP NPs under both normoxic (Figure 6f and Figure S51) and hypoxic conditions (Figure S52).

### 2.7 Release Capacity in the 3D MCTSs

As an in vitro model, 3D multicellular tumor spheroids (MCTSs) are commonly employed to simulate solid tumors. Subsequently, the ^1^O_2_ “charging” and “discharging” processes of PBDP NPs in 4T1 MCTSs was investigated using DCFH‐DA as a probe. As shown in Figure 6g and Figure S53, after 808 nm laser irradiation, the PBDP NPs group displayed green fluorescence due to photodynamic effects. In the dark, the green fluorescence in the 4T1 MCTSs continued to increase gradually over 30 s, which is attributed to the heat‐triggered re‐release of ^1^O_2_ by PBDP NPs. Therefore, PBDP NPs can be used as singlet oxygen battery for anti‐tumor therapy. Under light conditions, the resulting increase in cell temperature is attributed to the photothermal properties of the PBDP NPs, which in turn enhances their antitumor efficacy. After MCTSs were incubated with PBDP NPs under in dark or with irradiation, the MCTSs were then stained with calcium‐AM and PI. As shown in Figure 6h and Figure S54, the number of dead cells (indicated in red) in the 3D tumor spheroid model treated with PBDP NPs + L was significantly higher than that in other groups, suggesting that PBDP NPs + L exhibits the best performance in killing cancer cells.

### 2.8 In Vivo Imaging‐Guided Tumor Phototherapy

In vivo evaluation in healthy BALB/c mice showed no significant toxicity in body weight (Figure S55) or major organ histology (Figure S56), indicating the good biosafety of PBDP NPs. The tumor accumulation of PBDP NPs was assessed via NIR‐II Fluorescence. The fluorescence signal at the tumor site peaked at 24 h. (Figure 7a,b). Major organs and tumor of the mice were dissected at 72 h. As shown in Figure 7c, the potential for enrichment of PBDP NPs in tumor was further supported by the NIR‐II signal on tumors compared to the weak signals on heart, lung and kidneys. Fluorescence quantification also indicated that PBDP NPs were metabolized by the liver and spleen (Figure 7d). The biosafety of PBDP NPs was further evaluated through systematic hematological, biochemical, hemolysis, and biodistribution analyses (Figure S57). No significant abnormalities were observed in blood routine parameters (WBC, RBC, and PLT) or liver/kidney function markers (ALB, AST, ALT, BUN, and CREA) after treatment. Meanwhile, PBDP NPs induced negligible hemolysis (<5%) over the tested concentration range (Figure S58), indicating excellent hemocompatibility. As shown in Figure S59, NIR‐II fluorescence imaging revealed that PBDP NPs circulated in the bloodstream and were mainly distributed in the liver and spleen after intravenous injection. Blood fluorescence decay analysis showed a circulation half‐life of approximately 7.34 h, and the fluorescence signal gradually decreased over 72 h, suggesting progressive metabolism and clearance through the hepatosplenic system.

**FIGURE 7:**
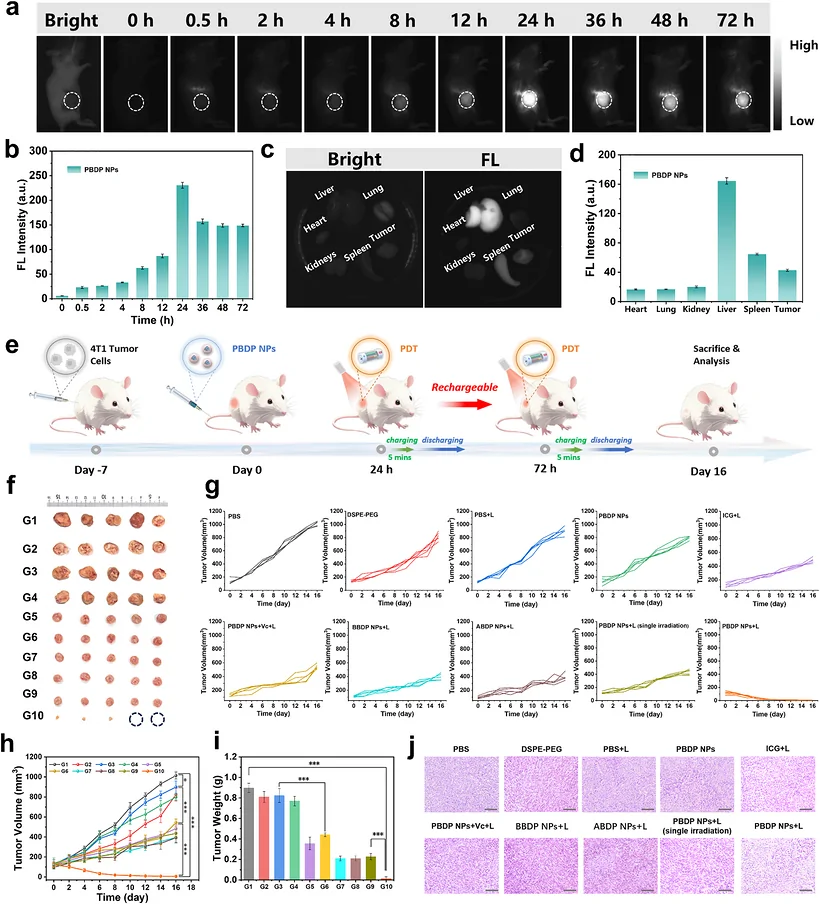
NIR‐II fluorescence images and anti‐tumor therapeutic effect in vivo of singlet oxygen battery. (a) NIR‐II fluorescence images of the 4T1 tumor‐bearing mouse at different time points after tail‐vein injection of PBDP NPs (100 ms exposure time). (b) The average NIR‐II fluorescence intensity of tumor tissues corresponding to (a). (c) NIR‐II fluorescence images of the main organs and tumor excised from the mouse after 72 h (25 ms exposure time). (d) Quantification of the fluorescence intensities of the excised tumors and organs. (e), Schematic illustration of the tumor treatment schedule. (f‐j) Monitoring data of various indicators during the treatment process(G1:PBS; G2:DSPE‐PEG; G3:PBS+L; G4:PBDP NPs; G5:ICG+L; G6:PBDP NPs+Vc+L; G7:BBDP NPs+L G8:ABDP NPs+L G9:PBDP NPs+L(single irradiation); G10:PBDP NPs+L): tumor photographs (f), tumor volume (g and h), tumor weight (i), and H&E staining of tumor sites (scale bar: 100 µm) (j) of different treated groups. Data are presented as the mean ± SD (n = 5). (*) p < 0.05 and (***) p < 0.001.

The in vivo antitumor efficacy of PBDP NPs was systematically evaluated in 4T1 tumor‐bearing mice with multiple control groups (Figure 7e). Infrared thermal imaging confirmed that PBDP NPs rapidly generated local hyperthermia under 808 nm laser irradiation, with the tumor temperature increasing to approximately 45°C within 5 min (Figure S60) Compared with all control treatments, PBDP NPs combined with repeated NIR irradiation (24 and 72 h post‐injection) achieved almost complete tumor suppression, with a tumor inhibition rate of 99% on day 16 (Figure 7f–j). In contrast, single irradiation after one injection resulted in only 57% tumor inhibition (Group 9), highlighting the enhanced therapeutic effect enabled by the rechargeable singlet oxygen storage‐release process. Mechanistic controls further demonstrated the unique contribution of the rechargeable singlet oxygen battery strategy. Compared with PBDP NPs + L, conventional photosensitizer ICG, ROS‐scavenged PBDP NPs + Vc, ^1^O_2_‐trapping‐deficient BBDP NPs, and another ^1^O_2_‐generating photosensitizer ABDP NPs showed significantly reduced tumor inhibition, confirming that sustained ^1^O_2_ release rather than photothermal effects or conventional PDT dominated the enhanced therapeutic efficacy. Notably, as shown in Figuer S61, free PBDP exhibited limited tumor inhibition (44%), further emphasizing the importance of nanoparticle formulation for effective tumor accumulation and treatment. In addition, tissue‐mimicking penetration experiments demonstrated that PBDP NPs maintained effective phototherapeutic damage through 6 mm tissue depth (Figure S62), supporting their potential for deep‐tissue tumor treatment. No obvious systemic toxicity was observed based on body weight monitoring, organ H&E staining, and biosafety evaluations (Figures S63–S66). Collectively, PBDP NPs achieved enhanced and sustained tumor suppression through rechargeable ^1^O_2_ storage–release combined with photothermal effects, while maintaining excellent biosafety and effective antitumor therapeutic potential.

## 3 Conclusion

In conclusion, we have developed a near‐infrared triggered rechargeable “singlet oxygen battery” based on PBDP NPs for sustainable phototherapeautic antitumor applications. By integrating aza‐BODIPY‐based photoconversion with 2‐pyridone as a reversible ^1^O_2_ carrier, PBDP NPs achieve efficient 808 nm‐excited generation, immediate storage via [4+2] cycloaddition, and sustained thermal release for up to 1 h after light cessation. Moreover, the system exhibits excellent rechargeability over multiple charging‐discharging cycles without performance degradation, while also providing concurrent photothermal conversion. In vivo NIR‐II imaging confirms effective tumor accumulation via the EPR effect, leading to significant tumor suppression through synergistic sustained PDT/PTT with minimal side effects. This work establishes a general and reusable ^1^O_2_ delivery paradigm, offering a promising strategy to address the critical challenges of tissue penetration, transient reactive oxygen species lifetime, and lack of recyclability in phototherapy.

## 4 Experimental Section

### 4.1 DFT Calculations

DFT calculations were carried out by the Gaussian 16 software. The B3LYP functional was adopted for all calculations in combination with the D3BJ dispersion correction. Geometry optimizations and singlet point energy calculations were performed at the level of B3LYP‐D3/6‐31G(d) in 1,4‐dioxane. The molecular orbitals were analyzed by Multiwfn.

### 4.2 Determination of Quantum Yield (QY) for NBDP, ABDP and PBDP

To measure the relative QY of NBDP, ABDP, and PBDP in 1,4‐Dioxane, ICG (13%) in Dimethyl sulfoxide (DMSO) was chosen as the reference. The quantum yield was calculated in the following formula:

$$\;QY_{sample}=QY_{ref}\;\frac{Slope_{sample}\;}{Slope_{ref}\;}{\left(\frac{n_{sample}}{n_{ref}}\right)}^{2}$$

Five different concentrations were chosen at or below OD 0.1 and the integrated fluorescence intensity was plotted against absorbance for NBDP, ABDP, and PBDP.

### 4.3 The Detection of ROS In Vitro

2′,7′‐dichlorodihydrofluorescein diacetate (DCFH‐DA) (1 mM 2 mL) and NaOH (0.01 M 8 mL) solution were mixed and reacted for 30 min in the dark. Then, PBS buffer (40 mL) was added to the mixture to obtain the DCFH working solution. The test solution was exposed to 808 nm laser, and the fluorescence spectra of the test solution were collected every minute at 525 nm (λex: 488 nm).

### 4.4 ESR Analysis

ESR analysis was performed to verify the generation of ^1^O_2_ under 808 nm irradiation by using TEMP as the spin trapping agent. ESR signals were obtained on irradiation of air‐saturated solution of TEMP (0.06 M) and PBDP NPs (40 µg/mL).

### 4.5 Photothermal Conversion Efficiency

For the purpose of evaluating the photothermal ability of nanoparticles, we discussed the effect of power density on conversion efficiency. The temperature changes were monitored by thermal camera. To measure the photothermal conversion efficiency, PBDP NPs (40 µg/mL) were exposed to 808 nm irradiation at 0.5 W/cm^2^ for 10 min, and then the solution was cooled down to room temperature. The temperature of the solution was recorded at an interval of 10 s during this process.

The photothermal conversion efficiencies (η) were measured according to the reported method:

$$\eta =\frac{hS\left(T_{Max}-T_{surr}\right)-Q_{Dis}}{I\left(1-10^{-A_{808}}\right)}$$

$$hS=\frac{mC}{\tau }$$

$$t=-\tau \ln\left(\theta \right)$$

$$\theta =\frac{T-T_{surr}}{T_{Max}-T_{surr}}$$

*η* represents the photothermal conversion efficiency, h denotes the heat transfer coefficient, S is the surface area of the container, *T_max_* and *T_Surr_* are the maximum steady state temperature and the environmental temperature, respectively. *Q_Dis_* represents heat dissipated from the laser mediated by the solvent and container, I means the laser power, A_808_ is the absorbance of polymers at 808 nm.

τ represents the system time constant of samples, *m* and *C* are the mass of the irradiated solution and the heat capacity of the solvent, respectively.

𝑡 is the time of the cooling period, and 𝜃 is the ratio of the real‐time temperature change in the cooling period to the maximum temperature change.

### 4.6 Determination of QYs for PBDP NPs

To measure the relative QYs of PBDP NPs in H_2_O, ICG (13%) in Dimethyl sulfoxide (DMSO) was chosen as the reference. The calculation procedure is similar to “Determination of quantum yield (QY) for NBDP, ABDP and PBDP”.

### 4.7 Measurement of ^1^O_2_ Generation

^1^O_2_ generation ability of PBDP NPs was examined by using ABDA, a commercial indicator that response to generated ^1^O_2_ by the decline of absorption intensity, as a specific probe. Typically, the PBDP NPs (40 µg/mL) and ABDA (40 µM) were dissolved in water. After that, the mixture was exposed to 808 nm irradiation (0.5 W/cm^2^). Measurements were carried out by recording the time‐depend decline of absorption intensity at ∼378 nm in a UV–vis spectrophotometer.

### 4.8 Co‐Staining Experiments Using Different Commerica Probes

MCF‐7 cells were cultured until they reached approximately 80% confluence. The culture medium was removed, and the cells were rinsed with phosphate‐buffered saline (PBS) to eliminate residual DMEM. Cells were incubated with PBDP NPs(40 µg/mL) for 12 h. Subsequently, cells were incubated for 30 min with DMEM‐based staining solutions containing either 500 nM MitoTracker Green or 75 nM LysoTracker Green, prepared from 0.5 mM and 75 µM stock solutions, respectively. After staining, the cells were washed twice with PBS and immediately subjected to bio‐imaging. Confocal laser scanning microscopy (CLSM) was used for image acquisition. PBDP‐NPs were excited at 559 nm, and their emission was collected using a GaAsP‐PMT detector in the range of 620–660 nm. MitoTracker Green and LysoTracker Green were excited at 488 nm, with emission collected between 500–550 nm.

### 4.9 MCTs Model of 4T1 Cells

The 3D multicellular tumor spheroid (MCTS) model of 4T1 cells was prepared as follows: 10 000 cells were added to each well of the Ultra‐Low Attachment 96‐Well Plate. On the tenth day, the cell spheres were basically formed.

### 4.10 Evaluation of Prolonged ^1^O_2_ Release of 4T1 MCTSs

After transferring cultivated tumor spheres to 96‐well plates, the incubate was replaced with 100 µL medium containing PBDP NPs (40 µg/mL), then cells were incubated for another 12 h, followed by incubation with DCFH‐DA (25 µM) for 30 min. Cells were treated with 808 nm irradiation (0.5 W/cm^2^). Finally, the fluorescence images of cells were obtained by an inverted fluorescence microscope, and recorded simultaneously with a thermal camera.

### 4.11 Animal Management

BALB/c mice (female, 4–6 weeks old) were obtained from the Experimental Animal Center of Shaanxi Normal University. All animal experiments were approved by the institutional animal care and use committee of Shaanxi Normal University (CE‐2024‐0012‐BG7). They were maintained under standard laboratory conditions with a ambient temperature of approximately 25 °C, relative humidity of 40–60%, and a 12‐h dark/light cycle. They had free access to standard diet and water. At the end of the experiment, the animals were euthanized by carbon dioxide inhalation.

### 4.12 Statistical Analysis

All quantitative data are presented as mean ± standard deviation (SD) from at least three independent biological replicates. Statistical analyses were performed using IBM SPSS Statistics. Comparisons between two groups were conducted using a two‐tailed Student's t‐test, while differences among multiple groups were evaluated by one‐way analysis of variance (ANOVA) followed by Tukey's multiple comparisons test. Statistical significance was defined as p < 0.05, with *p < 0.05, **p < 0.01, and ***p < 0.001 indicating significant, highly significant, and extremely significant differences, respectively.

## Author Contributions

**Yanli Tang**: conceptualization, methodology, formal analysis, supervision, funding acquisition, visualization, project administration, writing – review and editing. **Yuxin Ren**: conceptualization, methodology, software, data curation, investigation, validation, formal analysis, writing – original draft. **Ling Li**: methodology, investigation. **Xinyi Zhang**: methodology, investigation. **Meiqi Li**: investigation. **Junqing Wang**: investigation. **Mengpan Zhang**: investigation. **Jian Wang**: investigation.

## Funding

National Natural Science Foundation of China (Grant No. 22274095) and Fundamental Research Funds for the Central Universities (Grant No. GK202302004).

## Conflicts of Interest

The authors declare no conflicts of interest.

## Supporting information




**Supporting File**: advs78284‐sup‐0001‐SuppMat.docx.

## Data Availability

The data that support the findings of this study are available from the corresponding author upon reasonable request.
