# Supplementary material for: Near‐Infrared Triggered Rechargeable Singlet Oxygen Battery for Sustained Phototherapy: Fast Charging and Long‐Lasting Discharging
Source: Adv Sci (Weinh). 2026 Oct 12:e78284. Online ahead of print. doi: 10.1002/advs.78284 (PMC13658895; doi:10.1002/advs.78284)
Supplement: Supplementary file 1 [file ADVS-9999-e78284-s001.docx]

Supporting Information

**Near-Infrared Triggered Rechargeable Singlet Oxygen Battery for Sustained Phototherapy: Fast Charging and Long-Lasting Discharging**

*Yuxin Ren, Ling Li, Xinyi Zhang, Meiqi Li, Junqing Wang, Mengpan Zhang, Jian Wang, and Yanli Tang**

Yuxin Ren, Ling Li, Xinyi Zhang, Meiqi Li, Junqing Wang, Mengpan Zhang, Jian Wang, Yanli Tang

Key Laboratory of Applied Surface and Colloid Chemistry, Ministry of Education, Key Laboratory of Analytical Chemistry for Life Science of Shaanxi Province, School of Chemistry and Chemical Engineering, Shaanxi Normal University, Xi’an, Shaanxi Province 710119, P. R. China.

E-mail: yltang@snnu.edu.cn

**Table of Contents**

**1. Experimental Section**	**S-3**

**Materials and measurements**	**S-3**

**Synthesis of NBDP, ABDP, PBDP and BBDP**	**S-3**

**Preparation and characterization of nanoparticles**	**S-11**

**Cell culture**	**S-11**

**Cell cytotoxicity**	**S-11**

**Live-dead cell staining experiments**	**S-12**

**Detection of intracellular ROS**.	**S-12**

**Live-dead cell staining experiments of 4T1 MCTSs**	**S-12**

**Hemolysis assay**	**S-12**

**Establishment of 4T1 tumor models**	**S-13**

**In vivo NIR-II fluorescence imaging**	**S-13**

**In vivo antitumor efficacy**	**S-13**

**2. Supporting Figures**	**S-15**

**Figure S1-S66**	**S-15- S-46**

**3. Reference**	**S-46**

**1. Experimental Section**

**Materials and measurements.**

All reagents used in experiments were purchased from Bide Pharmatech Co. Ltd. (Shanghai, China), Aladdin Industrial Corporation (Shanghai, China) or J&K Chemical Ltd. (Beijing, China) and directly used without further purification. DSPE-PEG_2000_ was purchased from Ruixi Biological Technology Co. Ltd (Xi’an, China). Ultra-Low Attachment 96-Well Plates was purchased from Beyotime (Shanghai, China). Calcein AM/PI were was purchased from proteintech (Wuhan, China). Fetal bovine serum (FBS), Dulbecco’s Modified Eagle Medium (DMEM), Penicillin-Streptomycin (P/S) and 0.25% (1×) Trypsin were obtained from Gibco. 10 × phosphate buffer solution (PBS) was purchased from Sangon Biotech Co. Ltd. (Shanghai, China). Ultrapure water was collected from the Milli-Q reference system.

^1^H NMR and ^13^C NMR spectra were recorded on Bruker AVANCE lll 600MHz spectrometer or Bruker NEO 600MHz spectrometer. MALDI-TOF-MS was measured on a Bruker Autoflex speed spectrometer. The UV-vis absorption spectra were taken on a Shimadzu UV-2600 spectrophotometer. The fluorescence emission spectra were recorded on a HORIBA Fluorolog-QM spectrophotometer system. All NIR-II fluorescent images were performed by the NIROPTICS Infrared Region in vivo imaging system. The size distribution was measured on Brookhaven NanoBrook 90 PlusPALS system. SEM images were taken with HITACHI SU8220 scanning electron microscope. The absorbance for MTT analysis were performed on SpectraMax M5 microplate reader. Cell numbers were quantitated using Accuri C6 flow cytometer (Becton 110 Dickinson, Franklin Lakes, NJ). Confocal laser scanning microscopy images were recorded on Olympus FV1200.

**Synthesis of NBDP, ABDP, PBDP and BBDP**

**Compound 1**: Under the nitrogen atmosphere, the catalyst Tetrakis(triphenylphosphine) palladium (40 mg, 0.04 mmol) was added to a stirred mixture of (4-(dipheny lamino) phenyl) boronic acid (200 mg, 0.69 mmol), 2-Acetyl-5-bromothiophene (141 mg, 0.69 mmol) and Potassium carbonate (189 mg, 1.38 mmol) in Toluene (20 mL). The reaction mixture was stirred at 100 °C for 24 h. After the reaction solution was cooled to room temperature, toluene in the reaction solution was removed under reduced pressure. Then the mixed solution was extracted with dichloromethane and washed three times with saturated NaCl solution. Then the organic phase was dried with anhydrous Na_2_SO_4_ and concentrated under vacuum. The crude product was separated and purified by silica gel column chromatography (petroleum ether/ethyl acetate (V/V = 10/1)), and then dried in vacuo to obtain **compound 1**(143 mg, yield: 56%).^1^H NMR (600 MHz, DMSO-d6) δ 7.91 (d, *J* = 4.0 Hz, 1H), 7.66 (d, *J* = 8.7 Hz, 2H), 7.51 (d, *J* = 4.0 Hz, 1H), 7.37 – 7.33 (m, 4H), 7.12 (t, *J* = 7.4 Hz, 2H), 7.10 – 7.07 (m, 4H), 6.96 (d, *J* = 8.7 Hz, 2H), 2.52 (s, 3H).

**Compound 2**: 1,4-Dimethylnaphthalene (100 mg, 0.64 mmol) was dissolved in chloroform (10 mL) under nitrogen and exclusion of light. Bromine (102 mg, 0.64 mmol) was added over a period of 10 min at 0 °C. The reaction was warmed to room temperature and stirred for 2 h. The reaction was diluted with chloroform (20 mL) and washed three times with saturated NaCl solution. The organic layer was dried over Na_2_SO_4_ and purified by silica gel column chromatography (petroleum ether). and then dried in vacuo to obtain **compound 2** (130 mg, yield: 86%).^1^H NMR (600 MHz, Chloroform-d) δ 8.06 – 8.03 (m, 1H), 7.98 – 7.94 (m, 1H), 7.57 – 7.51 (m, 2H), 7.48 (d, *J* = 1.3 Hz, 1H), 2.77 (s, 3H), 2.63 (s, 3H).

**Compound 3**: Under the nitrogen atmosphere, the catalyst Tetrakis(triphenylphosphine) palladium (25 mg, 0.02 mmol) was added to a stirred mixture of 4-Formylphenylboronic acid (64 mg, 0.43 mmol), Compound 2 (100 mg, 0.43 mmol) and Potassium carbonate (297 mg, 2.15 mmol) in Toluene (20 mL). The reaction mixture was stirred at 100 °C for 24 h. After the reaction solution was cooled to room temperature, toluene in the reaction solution was removed under reduced pressure. The reaction was diluted with chloroform (20 mL) and washed three times with saturated NaCl solution. The organic layer was dried over Na_2_SO_4_ and purified by silica gel column chromatography (petroleum ether/ dichloromethane (V/V = 10/1)). and then dried in vacuo to obtain **compound 3** (72 mg, yield: 65%).^1^H NMR (600 MHz, Chloroform-d) δ 10.08 (s, 1H), 8.12 (dd, *J* = 7.5, 2.1 Hz, 1H), 8.05 (dd, *J* = 7.4, 2.1 Hz, 1H), 7.95 (d, *J* = 7.9 Hz, 2H), 7.59 (tt, *J* = 6.9, 5.2 Hz, 2H), 7.54 (d, *J* = 7.9 Hz, 2H), 7.23 (d, *J* = 12.0 Hz, 1H), 2.70 (s, 3H), 2.57 (s, 3H).

**Compound 4**: Under the nitrogen atmosphere, the catalyst Tetrakis(triphenylphosphine) palladium (78 mg, 0.07 mmol) was added to a stirred mixture of 4-Formylphenylboronic acid (200 mg, 1.33 mmol), 9-Bromoanthracene (343 mg, 1.33 mmol) and Potassium carbonate (919 mg, 6.65 mmol) in Toluene (20 mL). The reaction mixture was stirred at 100 °C for 24 h. After the reaction solution was cooled to room temperature, toluene in the reaction solution was removed under reduced pressure. Then the mixed solution was extracted with dichloromethane and washed three times with saturated NaCl solution. Then the organic phase was dried with anhydrous Na_2_SO_4_ and concentrated under vacuum. The crude product was separated and purified by silica gel column chromatography (petroleum ether/ethyl acetate (V/V = 10/1)), and then dried in vacuo to obtain **compound 4** (155 mg, yield: 41%). ^1^H NMR (600 MHz, Chloroform-d) δ 10.20 (s, 1H), 8.54 (s, 1H), 8.11 (d, J = 8.0 Hz, 2H), 8.07 (d, J = 8.5 Hz, 2H), 7.63 (d, J = 7.9 Hz, 2H), 7.57 (d, J = 8.8 Hz, 2H), 7.49 – 7.46 (m, 2H), 7.39 – 7.35 (m, 2H).

**Compound 5**: K_2_CO_3_ (345 mg, 2.50 mmol) was added to the reaction mixture of 4-(bromomethyl) benzaldehyde (100 mg, 0.50 mmol) and 2-pyridone (47 mg, 0.50 mmol) dissolved in acetone. After adding catalytic amount of 18-crown-6 (13 mg, 0.05 mmol), reaction was left to stir at room temperature. Then the mixed solution was extracted with dichloromethane and washed three times with saturated NaCl solution. Then the organic phase was dried with anhydrous Na_2_SO_4_ and concentrated under vacuum. The crude product was separated and purified by silica gel column chromatography (petroleum ether/ethyl acetate (V/V = 1/5)), and then dried in vacuo to obtain **compound 5** (72 mg, yield: 68%).^1^H NMR (600 MHz, Chloroform-d) δ 9.99 (s, 1H), 7.88 – 7.83 (m, 2H), 7.44 (d, *J* = 8.0 Hz, 2H), 7.37 (ddd, *J* = 9.0, 6.6, 2.1 Hz, 1H), 7.30 (dd, *J* = 6.8, 2.1 Hz, 1H), 6.67 – 6.63 (m, 1H), 6.21 (d, *J* = 1.4 Hz, 1H), 5.22 (s, 2H).

**Compound 6-R_1_:** **Compound 1** (284 mg, 0.77 mmol) and **Compound 3** (200 mg, 0.77 mmol) were mixed in 20 mL EtOH, and then NaOH solution (2.5 M, 10 mL) was added. The reaction solution was stirred at room temperature for 24 h. Then the mixed solution was extracted with dichloromethane and washed three times with saturated NaCl solution. Then the organic phase was dried with anhydrous Na_2_SO_4_ and concentrated under vacuum. The crude product was separated and purified by silica gel column chromatography (petroleum ether/ dichloromethane (V/V = 1/1)), and then dried in vacuo to obtain compound 6-R_1_ (230 mg, yield: 49%). ^1^H NMR (600 MHz, DMSO-d_6_) δ 8.15 – 8.12 (m, 1H), 8.07 – 8.04 (m, 1H), 7.93 (d, *J* = 15.6 Hz, 1H), 7.84 (d, *J* = 4.0 Hz, 1H), 7.76 – 7.73 (m, 2H), 7.58 (td, *J* = 7.9, 1.5 Hz, 2H), 7.56 – 7.54 (m, 2H), 7.50 (d, *J* = 15.6 Hz, 1H), 7.47 – 7.44 (m, 2H), 7.33 – 7.29 (m, 4H), 7.29 (d, *J* = 1.9 Hz, 1H), 7.16 – 7.13 (m, 4H), 7.11 – 7.05 (m, 4H), 2.71 (s, 3H), 2.61 (s, 3H).

**Compound 6-R_2_:** The synthesis method of **Compound 6-R_2_** is similar to that of **Compound 6-R_1_**. The crude product was separated and purified by silica gel column chromatography (petroleum ether/ dichloromethane (V/V = 1/1)), and then dried in vacuo to obtain **compound 6-R_2_** (155 mg, yield: 41%).^1^H NMR (600 MHz, Chloroform-d) δ 8.53 (s, 1H), 8.06 (d, *J* = 8.5 Hz, 2H), 8.02 (d, *J* = 15.6 Hz, 1H), 7.88 (s, 1H), 7.87 (d, *J* = 3.5 Hz, 2H), 7.68 (d, *J* = 8.8 Hz, 2H), 7.60 – 7.55 (m, 3H), 7.52 (d, *J* = 7.8 Hz, 2H), 7.48 (dd, *J* = 8.5, 6.4 Hz, 2H), 7.38 (dd, *J* = 8.7, 6.6 Hz, 2H), 7.31 (d, *J* = 1.4 Hz, 1H), 7.30 (t, *J* = 6.6 Hz, 4H), 7.15 (d, *J* = 7.9 Hz, 4H), 7.10 – 7.06 (m, 4H).

**Compound 6-R_3_:** The synthesis method of **Compound 6-R_3_** is similar to that of **Compound 6-R_1_**. The crude product was separated and purified by silica gel column chromatography (petroleum ether/ethyl acetate (V/V = 1/5)), and then dried in vacuo to obtain **compound 6-R_3_** (230 mg, yield: 47%).^1^H NMR (600 MHz, DMSO-d_6_) δ 8.31 (d, *J* = 4.1 Hz, 1H), 7.89 – 7.84 (m, 3H), 7.82 (dd, *J* = 6.8, 2.1 Hz, 1H), 7.73 – 7.67 (m, 3H), 7.61 (d, *J* = 4.0 Hz, 1H), 7.45 (ddd, *J* = 8.9, 6.6, 2.1 Hz, 1H), 7.36 (ddd, *J* = 8.4, 5.5, 3.2 Hz, 6H), 7.15 – 7.12 (m, 2H), 7.11 – 7.08 (m, 4H), 6.99 – 6.95 (m, 2H), 6.44 (dd, *J* = 9.2, 1.4 Hz, 1H), 6.27 (td, *J* = 6.7, 1.4 Hz, 1H), 5.14 (s, 2H).

**Compound 7-R_1_:** Compound 6-R_1_ (200 mg, 0.33 mmol), nitromethane (177 μL, 3.3 mmol) and diethylamine (340 μL, 3.3 mmol) were dissolved in ethanol (15 mL), and the reaction mixture was stirred at 90 °C for 12 h. After cooling to room temperature, the mixed solution was extracted with ethyl acetate and washed three times with saturated NaCl solution. Then the organic phase was dried with anhydrous Na_2_SO_4_ and concentrated under vacuum. The crude product was separated and purified by silica gel column chromatography (petroleum ether/ethyl acetate (V/V = 1/5)), and then dried in vacuo to obtain **compound 7-R_1_**(175 mg, yield: 79.0%).^1^H NMR (600 MHz, Chloroform-d) δ 8.10 (dd, *J* = 7.9, 1.7 Hz, 1H), 8.03 (dd, *J* = 7.7, 1.8 Hz, 1H), 7.66 (d, *J* = 4.0 Hz, 1H), 7.59 – 7.53 (m, 2H), 7.49 (d, *J* = 8.7 Hz, 2H), 7.39 – 7.32 (m, 4H), 7.32 – 7.27 (m, 4H), 7.23 – 7.19 (m, 2H), 7.16 – 7.11 (m, 4H), 7.11 – 7.03 (m, 4H), 4.92 (dd, *J* = 12.7, 6.5 Hz, 1H), 4.79 (dd, *J* = 12.7, 8.2 Hz, 1H), 4.28 (t, *J* = 7.2 Hz, 1H), 3.41 (qd, *J* = 16.6, 7.1 Hz, 2H), 2.67 (s, 3H), 2.53 (s, 3H).

**Compound 7-R_2_:** The synthesis method of **Compound 7-R_2_** is similar to that of **Compound 7-R_1_**. The crude product was separated and purified by silica gel column chromatography (petroleum ether/ethyl acetate (V/V = 1/1)), and then dried in vacuo to obtain **compound 7-R_2_** (52 mg, yield: 47%).^1^H NMR (600 MHz, Chloroform-d) δ 8.49 (s, 1H), 8.03 (d, *J* = 8.6 Hz, 2H), 7.70 (d, *J* = 4.0 Hz, 1H), 7.55 (d, *J* = 8.7 Hz, 2H), 7.51 (dd, *J* = 11.0, 8.1 Hz, 4H), 7.45 (d, *J* = 7.4 Hz, 2H), 7.40 (d, *J* = 7.6 Hz, 2H), 7.30 (q, *J* = 7.7, 6.6 Hz, 6H), 7.25 (d, *J* = 4.0 Hz, 1H), 7.14 (d, *J* = 7.9 Hz, 4H), 7.11 – 7.05 (m, 4H), 5.00 (dd, *J* = 12.6, 6.6 Hz, 1H), 4.89 (dd, *J* = 12.7, 8.2 Hz, 1H), 4.38 (s, 1H), 3.51 (d, *J* = 6.4 Hz, 1H), 3.47 (d, *J* = 7.6 Hz, 1H).

**Compound 7-R_3_:** The synthesis method of **Compound 7-R_3_** is similar to that of **Compound 7-R_1_**. The crude product was separated and purified by silica gel column chromatography (petroleum ether/ethyl acetate (V/V = 1/5)), and then dried in vacuo to obtain **compound 7-R_3_** (76 mg, yield: 68%).^1^H NMR (600 MHz, Chloroform-d) δ 7.60 (d, *J* = 4.0 Hz, 1H), 7.50 – 7.45 (m, 2H), 7.35 (ddd, *J* = 8.9, 6.6, 2.1 Hz, 1H), 7.31 – 7.28 (m, 3H), 7.26 (s, 6H), 7.19 (d, *J* = 4.0 Hz, 1H), 7.15 – 7.10 (m, 4H), 7.10 – 7.06 (m, 2H), 7.04 (d, *J* = 8.7 Hz, 2H), 6.72 – 6.68 (m, 1H), 6.20 (td, *J* = 6.7, 1.4 Hz, 1H), 5.12 (s, 2H), 4.84 (dd, *J* = 12.7, 6.4 Hz, 1H), 4.69 (dd, *J* = 12.7, 8.3 Hz, 1H), 4.23 – 4.15 (m, 1H), 3.37 – 3.25 (m, 2H).

**Compound 8-R_1_:** **C****ompound 7-R_1_** (80 mg, 0.12 mmol) and ammonium acetate (277 mg, 3.60 mmol) were mixed in n-butanol (20 mL), then reacted at 115 ºC for 48 h. After solvent was removed, the residue was filtered and washed with cool ethanol three times. The crude product was separated and purified by silica gel column chromatography (petroleum ether/ dichloromethane (V/V = 2/1)), and then dried in vacuo to obtain **compound 8-R_1_** (37 mg, yield: 37%).^1^H NMR (600 MHz, Chloroform-d) δ 8.18 (d, *J* = 7.8 Hz, 2H), 8.02 (d, *J* = 19.0 Hz, 2H), 7.91 (d, *J* = 8.4 Hz, 2H), 7.80 (d, *J* = 7.7 Hz, 2H), 7.73 (d, *J* = 8.4 Hz, 2H), 7.65 – 7.50 (m, 6H), 7.45 (dd, *J* = 19.0, 5.5 Hz, 4H), 7.30 (q, *J* = 10.7, 9.1 Hz, 17H), 7.20 (s, 2H), 7.17 – 6.94 (m, 14H), 2.62 (d, *J* = 18.0 Hz, 6H), 2.35 (d, *J* = 27.0 Hz, 6H).

**Compound 8-R_2_:** The synthesis method of **Compound 8-R_2_** is similar to that of **Compound 8-R_1_**. The crude product was separated and purified by silica gel column chromatography (petroleum ether/ dichloromethane (V/V = 2/1)), and then dried in vacuo to obtain **compound 8-R_2_** (55 mg, yield: 30%).^1^H NMR (600 MHz, DMSO-d_6_) δ 8.74 (d, *J* = 6.8 Hz, 2H), 8.64 – 8.59 (m, 2H), 8.49 (s, 2H), 8.39 (dd, *J* = 13.7, 7.8 Hz, 4H), 8.20 (t, *J* = 6.9 Hz, 4H), 8.07 (d, *J* = 8.7 Hz, 2H), 7.95 (s, 2H), 7.75 – 7.63 (m, 10H), 7.58 (d, *J* = 8.3 Hz, 4H), 7.49 (d, *J* = 7.9 Hz, 4H), 7.33 (q, *J* = 8.6, 7.9 Hz, 8H), 7.08 (d, *J* = 7.7 Hz, 8H), 7.01 (dd, *J* = 43.0, 8.2 Hz, 8H), 6.90 (s, 1H).

**Compound 8-R_3_:** The synthesis method of **Compound 8-R_3_** is similar to that of **Compound 8-R_1_**. The crude product was separated and purified by silica gel column chromatography (dichloromethane / Methanol (V/V = 20/1)), and then dried in vacuo to obtain **compound 8-R_3_** (60 mg, yield: 32%).^1^H NMR (600 MHz, DMSO-d_6_) δ 7.99 (d, J = 8.2 Hz, 2H), 7.88 – 7.79 (m, 3H), 7.66 – 7.63 (m, 2H), 7.60 (d, J = 4.0 Hz, 2H), 7.55 (d, J = 1.7 Hz, 2H), 7.51 – 7.42 (m, 4H), 7.37 – 7.28 (m, 12H), 7.08 (qt, J = 6.4, 2.0 Hz, 8H), 7.04 – 6.96 (m, 8H), 6.95 – 6.85 (m, 4H), 6.53 – 6.45 (m, 2H), 6.32 (td, J = 6.7, 1.4 Hz, 2H), 5.17 (s, 4H).

**NBDP:** Under the nitrogen atmosphe**re, Compound 8-R_1_** (37 mg, 0.03 mmol)_,_ DIPEA (53 μL, 0.30 mmol), and BF_3_•OEt_2_ (74 μL, 0.60 mmol) were dissolved in dichloromethane (10 mL), and the reaction mixture was stirred at room temperature for 12 h. The mixed solution was extracted with dichloromethane and washed three times with saturated NaCl solution. Then the organic phase was dried with anhydrous Na_2_SO_4_ and concentrated under vacuum. The crude product was separated and purified by silica gel column chromatography (petroleum ether/ dichloromethane (V/V = 2/1)), and then dried in vacuo to obtain **NBDP** (35 mg, yield: 90%).^1^H NMR (600 MHz, Chloroform-d) δ 8.13 – 8.10 (m, 2H), 8.06 – 8.03 (m, 2H), 7.58 (td, *J* = 7.8, 1.5 Hz, 4H), 7.50 (d, *J* = 8.6 Hz, 4H), 7.40 (d, *J* = 7.5 Hz, 4H), 7.34 (d, *J* = 8.0 Hz, 4H), 7.30 (t, *J* = 7.8 Hz, 11H), 7.21 (s, 2H), 7.17 – 7.11 (m, 10H), 7.09 (t, *J* = 7.3 Hz, 6H), 7.05 (d, *J* = 8.4 Hz, 4H), 2.70 (s, 6H), 2.56 (s, 6H). ^13^C NMR (101 MHz, Chloroform-d) δ 148.70, 147.30, 147.04, 146.94, 143.71, 138.05, 134.67, 133.11, 132.04, 131.96, 130.85, 130.08, 129.75, 129.47, 128.98, 128.88, 128.80, 127.13, 125.89, 125.41, 125.18, 125.13, 124.56, 123.81, 122.56, 53.44. MALDI-TOF-MS m/z: calcd for C_88_H_64_BF_2_N_5_S_2_^+^ 1304.47 [M + H] ^+^; found 1304.96 [M + H] ^+^.

**ABDP:** The synthesis method of **ABDP** is similar to that of **PBDP**. The crude product was separated and purified by silica gel column chromatography (petroleum ether/ dichloromethane (V/V = 2/1)), and then dried in vacuo to obtain **ABDP** (48 mg, yield: 88%).^1^H NMR (600 MHz, DMSO-d_6_) δ 8.61 (s, 2H), 8.47 (d, *J* = 7.9 Hz, 4H), 8.42 (d, *J* = 4.1 Hz, 2H), 8.08 (d, *J* = 8.6 Hz, 4H), 7.90 (s, 2H), 7.79 (d, *J* = 4.2 Hz, 2H), 7.74 (d, *J* = 8.6 Hz, 4H), 7.57 (dd, *J* = 15.2, 8.4 Hz, 8H), 7.40 – 7.35 (m, 12H), 7.26 – 7.21 (m, 4H), 7.16 (d, *J* = 7.4 Hz, 4H), 7.15 – 7.11 (m, 8H), 7.01 (d, *J* = 8.6 Hz, 4H). ^13^C NMR (101 MHz, Chloroform-d) δ 139.92, 136.19, 135.72, 134.90, 131.63, 131.41, 131.28, 130.01, 129.94, 129.91, 129.76, 129.53, 129.48, 129.16, 129.09, 128.86, 128.26, 127.10, 126.73, 126.61, 126.38, 125.69, 125.50, 125.27, 125.06, 124.73, 123.85, 122.54. MALDI-TOF-MS m/z: calcd for C_92_H_60_BF_2_N_5_S_2_ ^+^ 1348.44 [M + H] ^+^; found 1348.96 [M + H] ^+^.

**PBDP:** The synthesis method of **PBDP** is similar to that of **NBDP**. The crude product was separated and purified by silica gel column chromatography (dichloromethane / Methanol (V/V = 20/1)), and then dried in vacuo to obtain **PBDP** (55 mg, yield: 32%).^1^H NMR (600 MHz, DMSO-d_6_) δ 8.31 (d, *J* = 4.2 Hz, 2H), 8.14 – 8.05 (m, 4H), 7.87 – 7.82 (m, 2H), 7.73 (d, *J* = 4.2 Hz, 2H), 7.70 (d, *J* = 8.4 Hz, 2H), 7.66 (s, 2H), 7.49 (d, *J* = 2.1 Hz, 4H), 7.41 – 7.30 (m, 12H), 7.14 (dd, *J* = 19.3, 7.7 Hz, 12H), 6.99 (d, *J* = 8.5 Hz, 4H), 6.48 (d, *J* = 9.2 Hz, 2H), 6.32 (s, 2H), 5.19 (s, 4H). ^13^C NMR (101 MHz, Chloroform-d) δ 147.40, 147.16, 147.00, 139.63, 137.39, 129.88, 129.42, 128.17, 128.05, 127.01, 126.68, 125.13, 124.82, 124.59, 124.46, 123.81, 123.73, 123.50, 123.09, 122.54, 122.47, 121.15, 121.04, 106.45, 51.73. MALDI-TOF-MS m/z: calcd for C_76_H_54_BF_2_N_7_O_2_S_2_^+^ 1210.38 [M + H] ^+^; found 1210.37 [M + H] ^+^.

**Synthesis of BBDP**

**Compound 9:** **Compound 1** (200 mg, 0.54 mmol) and **4-Benzylbenzaldehyde** (106 mg, 0.54 mmol) were mixed in 20 mL EtOH, and then NaOH solution (2.5 M, 10 mL) was added. The reaction solution was stirred at room temperature for 24 h. Then the mixed solution was extracted with dichloromethane and washed three times with saturated NaCl solution. Then the organic phase was dried with anhydrous Na_2_SO_4_ and concentrated under vacuum. The crude product was separated and purified by silica gel column chromatography (petroleum ether/ dichloromethane (V/V = 1/1)), and then dried in vacuo to obtain compound 9 (210 mg, yield: 71%). ^1^H NMR (600 MHz, DMSO-d_6_) δ 8.30 (d, *J* = 4.1 Hz, 1H), 7.85 – 7.80 (m, 3H), 7.72 – 7.67 (m, 3H), 7.60 (d, *J* = 4.0 Hz, 1H), 7.38 – 7.32 (m, 6H), 7.31 – 7.28 (m, 2H), 7.27 – 7.24 (m, 2H), 7.22 – 7.18 (m, 1H), 7.15 – 7.11 (m, 2H), 7.11 – 7.08 (m, 4H), 6.97 (d, *J* = 8.8 Hz, 2H), 4.00 (s, 2H).

**Compound 10:** **Compound 9** (200 mg, 0.38 mmol), nitromethane (205 μL, 3.83 mmol) and diethylamine (397 μL, 3.83 mmol) were dissolved in ethanol (15 mL), and the reaction mixture was stirred at 90 °C for 12 h. After cooling to room temperature, the mixed solution was extracted with ethyl acetate and washed three times with saturated NaCl solution. Then the organic phase was dried with anhydrous Na_2_SO_4_ and concentrated under vacuum. The crude product was separated and purified by silica gel column chromatography (petroleum ether/ dichloromethane (V/V = 1/1)), and then dried in vacuo to obtain **compound 10**(190 mg, yield: 81%). ^1^H NMR (600 MHz, DMSO-d_6_) δ 7.90 (d, *J* = 4.1 Hz, 1H), 7.64 (d, *J* = 8.6 Hz, 2H), 7.48 (d, *J* = 4.0 Hz, 1H), 7.35 (t, *J* = 7.8 Hz, 4H), 7.27 (t, *J* = 6.7 Hz, 2H), 7.24 (d, *J* = 7.4 Hz, 2H), 7.19 (d, *J* = 7.5 Hz, 2H), 7.16 – 7.13 (m, 3H), 7.11 (d, *J* = 7.4 Hz, 2H), 7.08 (d, *J* = 7.9 Hz, 4H), 6.95 (d, *J* = 8.6 Hz, 2H), 4.96 (dd, *J* = 13.0, 5.7 Hz, 1H), 4.84 (dd, *J* = 13.0, 9.6 Hz, 1H), 4.00 – 3.93 (m, 1H), 3.87 (s, 2H), 3.45 (dd, *J* = 17.0, 7.5 Hz, 1H), 3.35 (d, *J* = 6.9 Hz, 1H).

**BBDP:** **Compound 10** (190 mg, 0.31 mmol) and ammonium acetate (721 mg, 9.36 mmol) were mixed in n-butanol (20 mL), then reacted at 115 ºC for 48 h. After solvent was removed, the residue was filtered and washed with cool ethanol three times to afford the crude product. Under the nitrogen atmosphere, the crude product described above, DIPEA (310 μL, 1.78 mmol), and BF_3_•OEt_2_ (220 μL, 1.78 mmol) were dissolved in dichloromethane (10 mL), and the reaction mixture was stirred at room temperature for 12 h. The mixed solution was extracted with dichloromethane and washed three times with saturated NaCl solution. Then the organic phase was dried with anhydrous Na_2_SO_4_ and concentrated under vacuum. The crude product was separated and purified by silica gel column chromatography (petroleum ether/ dichloromethane (V/V = 5/1)), and then dried in vacuo to obtain **BBDP** (90 mg, yield: 25%).

^1^H NMR (600 MHz, DMSO-d_6_) δ 8.29 (d, *J* = 4.1 Hz, 2H), 8.06 (d, *J* = 7.9 Hz, 4H), 7.70 (dd, *J* = 14.3, 6.2 Hz, 6H), 7.61 (s, 2H), 7.36 (t, *J* = 7.7 Hz, 8H), 7.32 (dd, *J* = 12.1, 7.7 Hz, 8H), 7.28 (d, *J* = 7.5 Hz, 4H), 7.26 – 7.19 (m, 4H), 7.14 (t, *J* = 7.4 Hz, 4H), 7.11 (d, *J* = 7.9 Hz, 6H), 6.98 (d, *J* = 8.3 Hz, 4H), 4.02 (s, 4H). ^13^C NMR (151 MHz, CDCl_3_) δ 148.60, 147.07, 142.36, 140.76, 130.47, 130.33, 129.49, 129.46, 129.29, 129.15, 128.99, 128.91, 128.60, 128.55, 127.00, 126.86, 126.28, 126.22, 125.19, 125.09, 123.74, 122.63, 122.42, 77.22, 77.01, 76.80, 41.82, 0.00.

**Preparation and characterization of** **nanoparticles^1^**

PBDP NPs: A mixture of PBDP (1 mg) and DSPE-PEG_2000_ (10 mg) were dissolved in THF. Under sonication conditions, the above solution was added dropwise into 10 mL of H_2_O. After mixing, the THF was removed by venting nitrogen. Then, the solution was filtered through a membrane filter (diameter = 0.22 μm) to obtain PBDP NPs. The morphology of PBDP NPs was characterized by SEM. The size distribution of PBDP NPs was measured on DLS.

ABDP NPs: A mixture of ABDP (1 mg) and DSPE-PEG_2000_ (10 mg) were dissolved in THF. Under sonication conditions, the above solution was added dropwise into 10 mL of H_2_O. After mixing, the THF was removed by venting nitrogen. Then, the solution was filtered through a membrane filter (diameter = 0.22 μm) to obtain ABDP NPs.

BBDP NPs: A mixture of BBDP (1 mg) and DSPE-PEG_2000_ (10 mg) were dissolved in THF. Under sonication conditions, the above solution was added dropwise into 10 mL of H_2_O. After mixing, the THF was removed by venting nitrogen. Then, the solution was filtered through a membrane filter (diameter = 0.22 μm) to obtain BBDP NPs.

**Cell culture**

MCF-7 (Human breast cancer cells) and HUVEC (Human umbilical vein endothelial cells) were cultured in DMEM containing 10% FBS and 1% penicillin-streptomycin at 37℃ with 5% CO_2_ in an incubator.

**Cell cytotoxicity^2^**

MCF-7 cells or HUVEC cells were seeded in 96-wells plates and incubated for 24 h, Subsequently, the incubate medium was replaced with 100 µL medium containing various concentrations of PBDP NPs. Then, cells were incubated for another 24 h. For the light group, cells were treated with 808 nm irradiation (0.5 W/cm^2^) for 5 min. Then, Methyl thiazolyl tetrazolium (MTT, 5 mg/mL, 10 μL) was added to each well, and the incubation was continued for 4 h. The medium was discarded, and DMSO (100 μL) was added into each well to dissolve the produced formazan. After shaking the plate for 10 min, the absorbance at 490 nm was measured via a microplate reader.

**Live-dead cell staining experiments^3^**

MCF-7 cells were seeded in 96-well plates. After incubating for 12 h, the incubate was replaced with 100 µL medium containing PBDP NPs (40 µg/mL), then cells were incubated for another 12 h. After different treatments, the old culture medium was discarded and washed with PBS for 3 times. For the light group, cells were treated with 808 nm irradiation (0.5 W/cm^2^) for 5 min. Then, the cells were incubated with medium containing Calcein AM (4 μM) and PI (9 μM) for 20 min. Finally, the fluorescence images of cells were obtained by an inverted fluorescence microscope.

**Detection of intracellular ROS**

DCFH-DA was applied for intracellular ROS generation. MCF-7 cells were incubated with PBDP NPs (40 μg/mL) for 12 hours, followed by incubation with DCFH-DA (25 μM) for 30 minutes. After that, cells were washed with PBS three times and irradiated for different times. The green fluorescence was observed using CLSM. (DCFH-DA: λex = 488 nm; λem = 525 nm).

**Live-dead cell staining experiments of 4T1 MCTSs**

After transferring cultivated tumor spheres to 96-well plates, the incubate was replaced with 100 µL medium containing PBDP NPs (40 µg/mL), then cells were incubated for another 12 h. After different treatments, the old culture medium was discarded and washed with PBS for 3 times. For the light group, cells were treated with 808 nm irradiation (0.5 W/cm^2^) for 5 min. Then, the cells were incubated with medium containing Calcein AM (4 μM) and PI (9 μM) for 40 min. Finally, the fluorescence images of cells were obtained by an inverted fluorescence microscope.

**Hemolysis assay**

Fresh mouse red blood cells (RBCs) were collected and washed three times with PBS by centrifugation (3000 rpm, 5 min) to remove plasma components. The obtained RBCs were diluted with PBS to prepare a 2% (v/v) RBC suspension. Then, 0.5 mL of RBC suspension was mixed with 0.5 mL of PBDP NPs at different concentrations and incubated at 37 °C for 4 h. PBS and deionized water were used as negative and positive controls, respectively. After incubation, all samples were centrifuged at 3000 rpm for 5 min, and the absorbance of the supernatant at 540 nm was measured using a microplate reader. The hemolysis ratio was calculated according to the following equation:

Hemolysis ratio (%) =$\frac{A_{s}-A_{n}}{A_{P}-A_{n}}\times100\%$

where $A_{s}$, $A_{n}$, and $A_{p}$represent the absorbance values of the sample, PBS control, and dd H_2_O control, respectively.

**Establishment of 4T1 tumor models**

To establish the tumor models, 4T1 tumor cells (2.5 × 10^6^ cells dissolved in PBS) were subcutaneously implanted into mice. 4T1-tumor bearing mice were used for in vivo animal experiments upon tumors reaching about 100 mm^3^.

**In vivo NIR-II fluorescence imaging**

4T1 tumor-bearing mice were intravenous injected with PBDP NPs (100 μL, 200 μg/mL). In vivo NIR-II fluorescence images were recorded at different time points by the InGaAs CCD camera equipped with a 1000 nm long-pass filter (0.3 W/cm^2^).

**In vivo antitumor efficacy**

When the tumor volume reached approximately 100 mm³, the 4T1 tumor-bearing mice were randomly divided into different treatment groups (n = 5 per group) including PBS, DSPE-PEG, PBS + L, PBDP NPs, ICG + L, PBDP NPs + Vc + L, BBDP NPs + L, ABDP NPs + L, PBDP NPs + L (single irradiation), and PBDP NPs + L (repeated irradiation). The corresponding formulations were intravenously injected via the tail vein. For all laser irradiation groups (except for the single irradiation group), tumor sites were irradiated with an 808 nm laser (0.5 W/cm^2^) for 5 min at 24 h post-injection, followed by a second irradiation under the same conditions at 72 h post-injection. For the single-irradiation group, only one laser treatment was applied at 24 h post-injection. During the treatment period, tumor volumes and body weights were recorded every two days. The tumor volume was calculated using the formula: V = (length × width^2^)/2, At the end of the treatment period, mice were euthanized by CO₂ inhalation, and tumors and major organs (heart, liver, spleen, lung, and kidney) were collected. The excised tumors were photographed and weighed. Tumor tissues and major organs were fixed in 4% paraformaldehyde for subsequent hematoxylin and eosin (H&E) staining.

For in vivo photothermal imaging, tumor-bearing mice were treated with PBDP NPs and irradiated with an 808 nm laser (0.5 W cm⁻²) for 5 min, and the temperature changes at the tumor site were recorded using an infrared thermal imaging camera.

**2. Supporting Figures**

**Figure S1.** ^1^H-NMR spectrum of **compound 1** in DMSO-d_6_.

**Figure S2.** ^1^H-NMR spectrum of **compound 2** in CDCl_3_.

**Figure S3.** ^1^H-NMR spectrum of **compound 3** in CDCl_3_.

**Figure S4.** ^1^H-NMR spectrum of **compound 4** in CDCl_3_.

**Figure S5.** ^1^H-NMR spectrum of **compound 5** in CDCl_3_.

**Figure S6.** ^1^H-NMR spectrum of **compound 6-R_1_** in DMSO-d_6_.

**Figure S7.** ^1^H-NMR spectrum of **compound 6-R_2_** in CDCl_3_.

**Figure S8.** ^1^H-NMR spectrum of **compound 6-R_3_** in DMSO-d_6_.

**Figure S9.** ^1^H-NMR spectrum of **compound 7-R_1_** in CDCl_3_.

**Figure S10.** ^1^H-NMR spectrum of **compound 7-R_2_** in CDCl_3_.

**Figure S11.** ^1^H-NMR spectrum of **compound 7-R_3_** in CDCl_3_.

**Figure S12.** ^1^H-NMR spectrum of **compound 8-R_1_** in CDCl_3_.

**Figure S13.** ^1^H-NMR spectrum of **compound 8-R_2_** in DMSO-d_6_.

**Figure S14.** ^1^H-NMR spectrum of **compound 8-R_3_** in DMSO-d_6_.

**Figure S15.** ^1^H-NMR spectrum of **NBDP** in CDCl_3_.

**Figure S16.** ^1^H-NMR spectrum of **ABDP** in DMSO-d_6_.

**Figure S17.** ^1^H-NMR spectrum of **PBDP** in DMSO-d_6_.

**Figure S18.** ^13^C-NMR spectrum of **NBDP** in CDCl_3_.

**Figure S19.** ^13^C-NMR spectrum of **ABDP** in CDCl_3_.

**Figure S20.** ^13^C-NMR spectrum of **PBDP** in CDCl_3_.

**Figure S21.** MALDI-TOF-MS Spectrum of **NBDP**.

**Figure S22.** MALDI-TOF-MS Spectrum of **ABDP**.

**Figure S23.** MALDI-TOF-MS Spectrum of **PBDP**.

**Figure S24.** Integrated emission intensities of NBDP, ABDP, PBDP, and ICG as a function of 808 nm absorbance, respectively.

**Figure S25**. (a-c) Time-dependent SOSG fluorescence emission spectra in the presence of NBDP, ABDP, and PBDP, respectively, during 808 nm laser irradiation for 0-5 min. (d) Changes in SOSG fluorescence intensity at 535 nm (F-F_0_) for the three molecules as a function of irradiation time. After 5 min of charging, the laser was switched off and the samples were maintained in the dark. (e-g) Time-dependent SOSG fluorescence emission spectra of the NBDP, ABDP, and PBDP systems, respectively, during the subsequent dark-discharge process for 0-60 min. (h) Changes in SOSG fluorescence intensity at 535 nm (F-F_0_) during dark discharge, reflecting the relative amounts of stored singlet oxygen subsequently released by the three molecules. The SOSG fluorescence spectra were recorded with excitation at 488 nm. Data are presented as the mean ± SD (n = 3).

**Figure S26.** (a) SOSG fluorescence spectra recorded during 808 nm laser irradiation (0–5 min). (b) Time-dependent SOSG fluorescence spectra during dark incubation (0–60 min).

**Figure S27**. Long-term colloidal stability of PBDP NPs in PBS. (a) Representative hydrodynamic size distributions of PBDP NPs dispersed in PBS at different time points (0, 0.5, 2, 4, 8, 12, 24, 36, 48, and 72 h). (b–d) Time-dependent changes in hydrodynamic diameter (b), PDI (c), and zeta potential (d) of PBDP NPs in PBS over 72 h.

**Figure S28**. Long-term colloidal stability of PBDP NPs in serum-containing culture medium. (a) Representative hydrodynamic size distributions of PBDP NPs dispersed in serum-containing culture medium at different time points (0, 0.5, 2, 4, 8, 12, 24, 36, 48, and 72 h). (b–d) Time-dependent changes in hydrodynamic diameter (b), PDI (c), and zeta potential (d) of PBDP NPs in serum-containing culture medium over 72 h.

**Figure S29.** Normalized (a) absorption spectra and (b) emission spectra of nanoparticles in H_2_O.

**Figure S30.** (a) UV-Vis absorption spectra of PBDP at different concentrations. (b) Linear fitting curve of absorbance versus concentration for PBDP at 855 nm.

**Figure S31.** Integrated emission intensities of PBDP NPs as a function of 808 nm absorbance, respectively.

**Figure S32.** The fluorescence spectra of DCF in the presence of PBDP after irradiation at different times.

**Figure S33.** The absorbance of ABDA probe at 378 nm in the presence of PBDP NPs with or without irradiation.

**Figure S34.** Degradation of ABDA in the presence of MB (a) and ICG (b) with or without light (660 nm, 0.1 W/cm^2^; 808 nm, 0.5 W/cm^2^).

**Figure S35.** (a) UV-Vis absorption changes of DPBF in water with and without PBDP NPs (40 μg/mL). (b) Comparative evaluation of the ^1^O_2_ generation ability of the same type of photosensitizers after multiple light-dark cycles.

**Figure S36.** Synthetic route of **BBDP**.

**Figure S37.** ^1^H-NMR spectrum of **compound 9** in DMSO-d_6_.

**Figure S38.** ^1^H-NMR spectrum of **compound 10** in DMSO-d_6_.

**Figure S39.** ^1^H-NMR spectrum of **BBDP** in DMSO-d_6_.

**Figure S40.** ^13^C-NMR spectrum of **BBDP** in CDCl_3_.

**Figure S41**. (a) Schematic illustration of the preparation of BBDP NPs by self-assembly of BBDP and DSPE-PEG_2000_. (b) Photothermal heating and cooling curves of BBDP NPs under laser irradiation and linear fitting of −ln(θ) versus time. (c) SOSG fluorescence spectra of BBDP NPs under laser irradiation for 0–5 min. (d) SOSG fluorescence spectra of BBDP NPs during dark treatment for 0-60 min after laser irradiation.

**Figure S42**. Quantitative analysis of the charging and discharging behavior of singlet oxygen battery. (a) Changes in the normalized absorbance of PBDP NPs at 878 nm during 808 nm laser irradiation for 5 min, followed by dark discharge for 60 min. (b) Time-dependent apparent conversion percentage of PBDP NPs during laser irradiation. (c) Time-dependent absorption recovery percentage of PBDP NPs during the subsequent dark-discharge process. Data are presented as the mean ± SD (n = 3).

**Figure S43**. (a) Time-dependent fluorescence spectra of SOSG in the presence of PBDP NPs during 5 min of light irradiation. (b) Fluorescence spectra of SOSG alone under the same irradiation conditions.

Figure S44. Absorption recovery percentages of PBDP NPs after each of ten consecutive charging-discharging cycles.

**Figure S45**. (a) Time-dependent UV–vis absorption spectra of ABDA in the presence of PBDP NPs at 43 °C during 808 nm laser irradiation for 5 min and subsequent dark incubation for up to 1 h. (b) Time-dependent UV–vis absorption spectra of ABDA alone under the same conditions. (c) Quantitative changes in ABDA absorbance at 378 nm, expressed as A_0_-A, for ABDA alone and ABDA + PBDP NPs during laser irradiation and subsequent dark conditions. Data are presented as the mean ± SD (n = 3).

**Figure S46.** NIR-II imaging of MCF-7 cells after incubating with PBDP NPs at different time points (808 nm, 0.3 W/cm^2^).

**Figure S47**. Cell viability of MCF-7 cells treated with different concentrations of PBDP NPs in the presence or absence of Vc under laser irradiation. Data are presented as mean ± SD (n=3).

**Figure S48.** The fluorescence quantification to figure 6e.

**Figure S49.** Confocal laser scanning microscopy images of MCF-7 cells stained with DCFH-DA under hypoxic tumor microenvironment and corresponding fluorescence quantification.

**Figure S50**. Intracellular ROS generation in MCF-7 cells detected by DCFH-DA staining and flow cytometry.

**Figure S51.** The fluorescence quantification to figure 6f.

**Figure S52.** Calcein-AM (green, live cells)/propidium iodide (PI; red, dead cells) staining of MCF-7 cells treated with PBDP NPs without or with laser irradiation under hypoxic tumor microenvironment (scale bar: 100 μm) and corresponding fluorescence quantification.

**Figure S53.** (a) The ROS fluorescence quantification and (b) photothermal temperature quantification to figure 6g.

**Figure S54.** The fluorescence quantification to figure 6h.

**Figure S55.** Safety profiles of PBDP NPs in BALB/c mice. Changes in body weight during 20 days with various treatments.

**Figure S56.** Safety profiles of PBDP NPs in BALB/c mice. Hematoxylin and eosin (H&E) staining for the main organs extracted from the mice at the end of the treatments. Scale bars: 100 μm.

**Figure S57.** Changes in WBC(a), RBC(b), PLT(c), ALB(d), AST(e), ALT (f), BUN (g), and CREA(h) after 20 days with various treatments. Data are presented as the mean ± SD (n = 3).

**Figure S58.** Relative hemolysis rate of red blood cells treated with different concentrations of PBDP NPs by using ddH_2_O as a positive control.

**Figure S59**. (a) NIR-II images obtained from the ventral position at different times after PBDP NPs intravenous injection (𝜆_ex_: 808 nm, 0.3 W/cm^2^, 1000 nm long-pass filter, 100 ms). b) NIR-II images of blood samples at different times during 72 h (25 ms exposure time). c) The corresponding NIR-II signal intensity of (b). The half-time (T_1/2_) was calculated to be ∼7.34 h. (d) NIR-II images of dissected organs at 72 h after PBDP NPs intravenous injection (25 ms exposure time). (e) The corresponding NIR-II signal intensity of (d). Data are presented as the mean ± SD (n = 3).

**Figure S60**. Infrared thermal images and corresponding temperature changes at the tumor sites of tumor-bearing mice during 808 nm laser irradiation.

**Figure S61.** Tumor growth curves after treatment with free PBDP after intravenous injection and corresponding photographs of excised tumors.

**Figure S62**. (a) Schematic illustration of laser irradiation of tumor samples through chicken muscle tissue with different thicknesses (0, 2, 4, 6, 8, 10, and 12 mm). Tumor samples were collected 12 h after tail-vein injection of PBDP NPs, and the irradiation schedule was consistent with the in vivo therapeutic protocol. (b) Representative H&E staining images of tumor samples after treatment under different chicken muscle tissue thicknesses. (scale bar: 100 μm)

**Figure S63.** Monitoring data of body weights during the treatment process.

**Figure S64.** Body photographs during the treatment process.

**Figure S65.** Representative photographs of the main organs extracted from the mice in the various groups at the end of the treatments.

**Figure S66.** Hematoxylin and eosin (H&E) staining for the main organs extracted from the mice in the various groups at the end of the treatments. Scale bars: 100 μm.

**3. Reference**

1.	J. Yan, X. Ma, D. Liang, et al., “An Autocatalytic Multicomponent DNAzyme Nanomachine for Tumor-Specific Photothermal Therapy Sensitization in Pancreatic Cancer,” *Nature Communications* 14 (2023): 6905, https://doi.org/10.1038/s41467-023-42740-2.

2.	Y. Chen, T. Xiong, Q. Peng, et al., “Self-reporting photodynamic nanobody conjugate for precise and sustainable large-volume tumor treatment,” *Nature Communications* 15 (2024): 6935, https://doi.org/10.1038/s41467-024-51253-5.

3.	B. Bao, Q. Yuan, Q. Feng, et al., “Regulating Signal Pathway Triggers Circular Reactive Oxygen Species Production to Augment Oxidative Stress with Enzyme-Activated Nanoparticles,” *CCS Chemistry* 6 (2024): 1-9, https://doi.org/10.31635/ccschem.023.202303370.

4.	W. Xiao, P. Wang, C. Ou, et al., “2-Pyridone-functionalized Aza-BODIPY photosensitizer for imaging-guided sustainable phototherapy,” *Biomaterials* 183 (2018): 693-708, https://doi.org/10.1016/j.biomaterials.2018.08.034.

5.	I. S. Turan, D. Yildiz, A. Turksoy, G. Gunaydin, and E. U. Akkaya, “A Bifunctional Photosensitizer for Enhanced Fractional Photodynamic Therapy: Singlet Oxygen Generation in the Presence and Absence of Light,” *Angewandte Chemie International Edition* 55 (2016): 2875-2878, https://doi.org/10.1002/anie.201511345.
